# Asymmetric Isochalcogenourea-Catalyzed Synthesis of 3,4-Dihydropyrans via (4+2)-Cycloadditions of Ethyl But-3-ynoate with Michael Acceptors

**DOI:** 10.1055/a-2659-8340

**Published:** 2025-08-18

**Authors:** Mario Hofer, Magdalena Piringer, Anna Scheucher, Lukas S. Vogl, Mario Waser

**Affiliations:** a Institute of Organic Chemistry, Johannes Kepler University Linz, Altenbergerstrasse 69, 4040 Linz, Austria

**Keywords:** Organocatalysis, Lewis Bases, Isochalcogenoureas, Cycloadditions, Alkynes, Heterocycles

## Abstract

We herein report the use of ethyl but-3-ynoate as a C2 building block for asymmetric (4+2)-heterocycloadditions with various Michael acceptors. Upon using chiral isochalcogenoureas as Lewis base catalysts, these reactions can be carried out with good to excellent control of the regioselectivity, diastereoselectivity, and enantioselectivity.

## Graphical abstract

**Figure:**
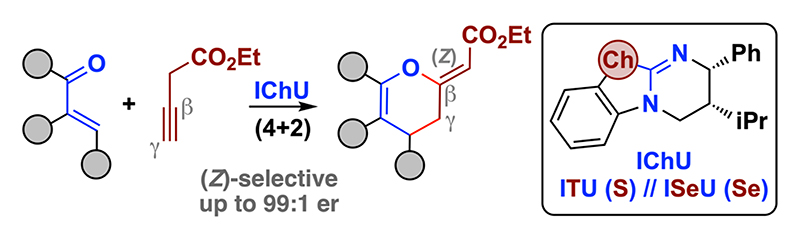

The pyran scaffold is a motif that is frequently encountered in a broad variety of biologically active (natural) products as well as building blocks and intermediates employed for the synthesis of pharmaceutically relevant molecules.^1–3^ Amongst different synthesis strategies, formal (4+2)-heterocycloadditions stand out, allowing for the direct syntheses of these heterocyclic 6-ring motifs even in an asymmetric manner.^4–8^ Our group has recently contributed to the field by establishing chiral isochalcogenoureas (IChUs)^9–11^ as Lewis base catalysts for highly enantioselective (4+2)-cycloadditions of allenoates **1** with different Michael acceptors (Scheme 1A).^7^ Remarkably, so far this protocol was found to be rather generally applicable for a broad variety of different allenoates and Michael acceptors. In all cases, we observed the highly selective formation of 3,4-dihydropyrans (DHPs) containing a (Z)-configurated exocyclic double bond. Mechanistically, these reactions are initiated by addition of the IChU to the β-carbon of the allenoate. The resulting dipolar betaine species **Int-I** then serves as a chiral C2 synthon reacting via its γ- and β-carbons in the formal (4+2)-cycloadditions with the Michael acceptors.^7^ It should be emphasized that the strict preference of the (4+2)-cycloaddition pathway observed for IChUs is in sharp contrast to analogous allenoate reactions catalyzed by alternative Lewis bases such as phosphines or amines.^5,6^ Based on these previous results, we hypothesized that we should be able to expand this concept beyond the classical allenoates **1** to alternative starting materials. We have very recently already shown that allenylsulfones and propargylic sulfones can be successfully used too (the later species undergo in situ isomerization to allene species under basic conditions).^8^

Inspired by these latest results, which showed that alkynes are suited C2 synthons under IChU catalysis as well, we were now wondering whether but-3-ynoates such as compound **2** may be suited as well. It is well known that such simple alkynes tend to isomerize to allenes under basic conditions and that they can be used for various cycloadditions (mainly (2+2) or (3+2)).^12–16^

However their successful utilization for asymmetric (4+2)-heterocycloadditions with Michael acceptors has so far, to the best of our knowledge, not been established. Thus, we have now investigated the suitability of the ethyl but-3-ynoate **2** to undergo analogous IChU-catalyzed cycloadditions as established for allenoates **1** before (Scheme 1B; formation of **Int-I** will again be the key step hereby). We were especially curious to see if alkyne **2** allows for the same highly stereoselective and regioselective (4+2) reaction pathway as **1** and if a comparably high generality tolerating different acceptors can be achieved.

We started by testing the reaction of **2** with the barbiturate-based acceptors **3** (Table 1). This transformation was chosen for our first proof-of-concept screening as we recently observed that the (4+2)-cycloadditions of **3** with allenoates **1** (giving products **9**) are the least enantioselective ones amongst the so far developed applications (we usually obtained er values of 95:5 up to >99:1 for acceptors **4**-**8**^7^ but surprisingly compounds **3** only allowed for around 85:15 er in most cases^7b^). Thus, we considered this a well-suited test reaction as differences in performance between allenoates **1** and alkyne **2** should be readily observable in both “directions” hereby. As catalysts we used the isothiourea (ITU) HyperBTM and the analogous isoselenourea (IseU) HyperSeBTM, as these were found to be the catalysts of choice for all our allenoate cycloadditions so far.^7,8^

First, we reacted an excess of alkyne **2** with the acceptor **3a** in the presence of 10 mol% of Hyper(Se)BTM and 1 eq. of K_2_CO_3_ in toluene at 120 °C (entries 1, 2). These conditions are based on our recently developed allenoate protocol^7^ and the only difference is the addition of the base, which we added to ensure an in situ isomerization of alkyne **2** to the corresponding allenoate (it should be emphasized that freshly prepared **2** already contains small amounts of the allenoate **1**, substantiating that this isomerization is a rather favorable process^12^). These first experiments confirmed the feasibility of this strategy, delivering the targeted (Z)-configurated (4+2)-cycloaddition product **9a** in good yields and with enantiomeric ratios comparable to the optimized allenoate protocol (for this product we obtained 86:14 er with HyperSeBTM before^7b^). In line with our previous observations, the ISeU catalyst was slightly better suited than the ITU. When reducing the amount of alkyne **2** we observed a pronounced impact of the base. While K_2_CO_3_ did not allow for full conversion anymore when using 2.5 eq. **2** (entry 3), Cs_2_CO_3_ performed better (entry 4), and Et_3_N gave the best yield, accompanied with a marginally increased enantioselectivity (entry 5). Upon using Et_3_N it was further possible to reduce the amount of **2** to 1.5 eq. without affecting yield and er (entries 6, 7). Knowing that this elevated temperature and toluene are crucial for these reactions when starting from allenoates,^7b^ we did not carry out any detailed solvent or temperature screening anymore after we identified these Et_3_N-mediated conditions. We next tested the suitability of acceptors **3b-e** and observed in general good yields and reasonable enantioselectivities for products **9**, lying in the same range, or sometimes even higher, as for allenoates **1** (entries 8-15). We have recently already shown that different sulfone-containing alkynes are well tolerated for such cycloadditions^7^ and we also know that the analogous allenoate-based transformations are in general pretty tolerant to different ester functionalities and to some extent also substituents in the α- or γ-positions.^7^ To get further insights we also tested a more advanced alternative ethyl butynoate **2** containing an α-methyl and a γ-phenyl substituent for the reaction with **3**, but unfortunately the method hereby came to its limit, resulting in a rather messy outcome with no selective product formation at all.

Having shown that alkyne **2** can be used analogously to allenoates **1** for reactions with acceptors **3**, we also tested the cycloadditions between **2** and the alternative acceptors **4, 6, 7**, and **8** (Scheme 2). In neither case did we carry out any real optimization but we used catalyst loadings, solvents, reaction temperatures, and reaction times as optimized for the corresponding allenoate approaches. Encouragingly, the yields and enantioselectivities for products **10**-**13** were very much comparable to our allenoate approaches, demonstrating the generality of this approach. More specifically, products **10** were obtained in up to 98:4 er, except for the NO_2_-containing **10c**, which was also not tolerated that well when using allenoates.^7a^ The other products **11**-**13** were all accessed with almost perfect enantioselectivities, but the yields were sometimes found a bit lower as compared to the allenoate protocols. Interestingly, when using the preformed o-quinone methides **7** (Scheme 2C), the reactions also performed well in the absence of base.

In conclusion, we could show that alkynes **2** represent interesting starting materials which can be successfully used as alternatives to preformed allenoates **1** for asymmetric (4+2)-heterocycloadditions under chiral IChU catalysis. This broadens the applicability of this methodology giving various highly functionalized dihydropyrans straightforwardly.

## Supplementary Material

Supporting Info

## Figures and Tables

**Scheme 1:**
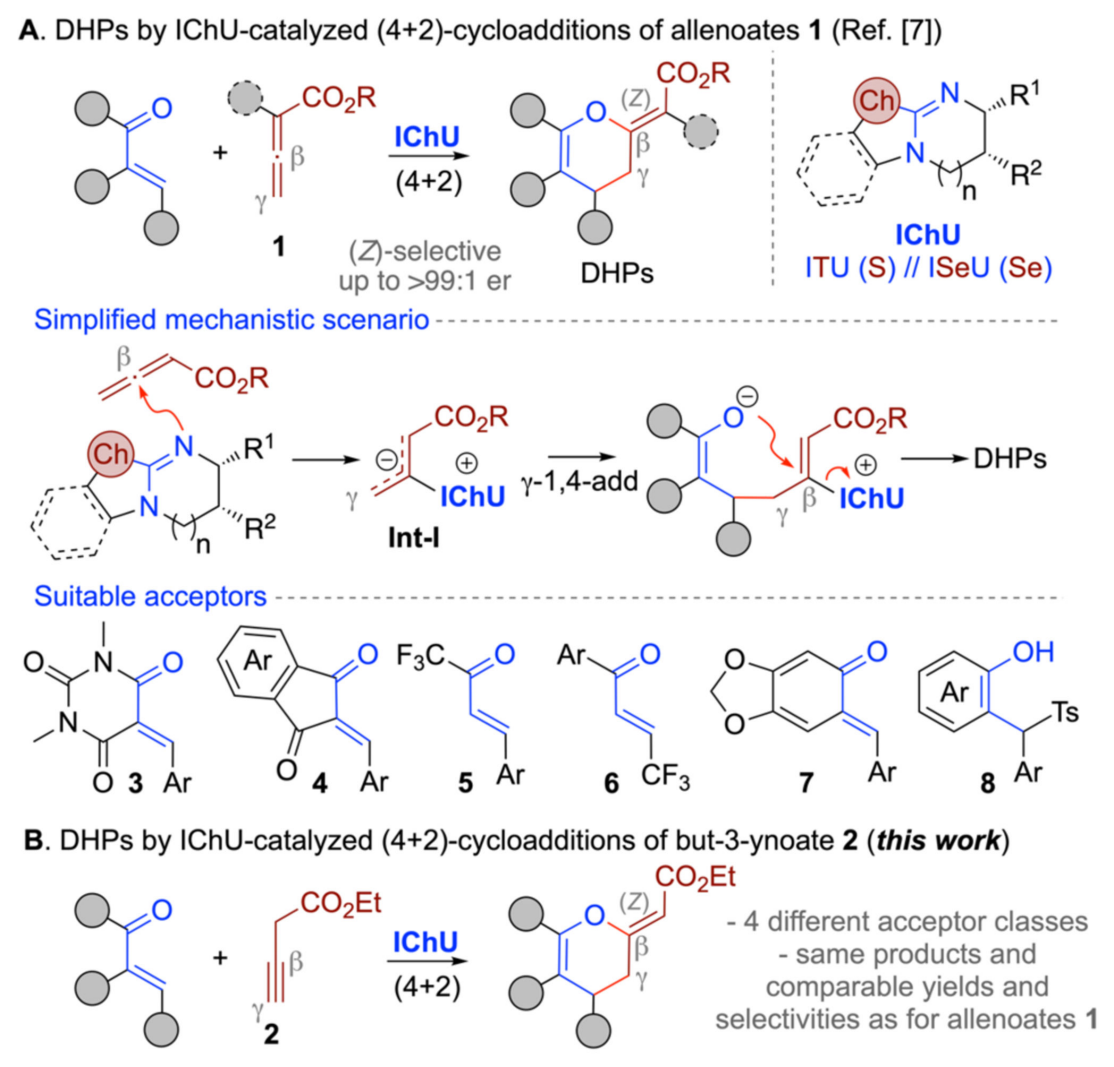
Our recently introduced IChU-catalyzed (4+2)-cycloadditions of allenoates **1** to access dihyropyranes (DHPs) (A) and the herein investigated use of alkynes **2** for analogous transformations.

**Scheme 2:**
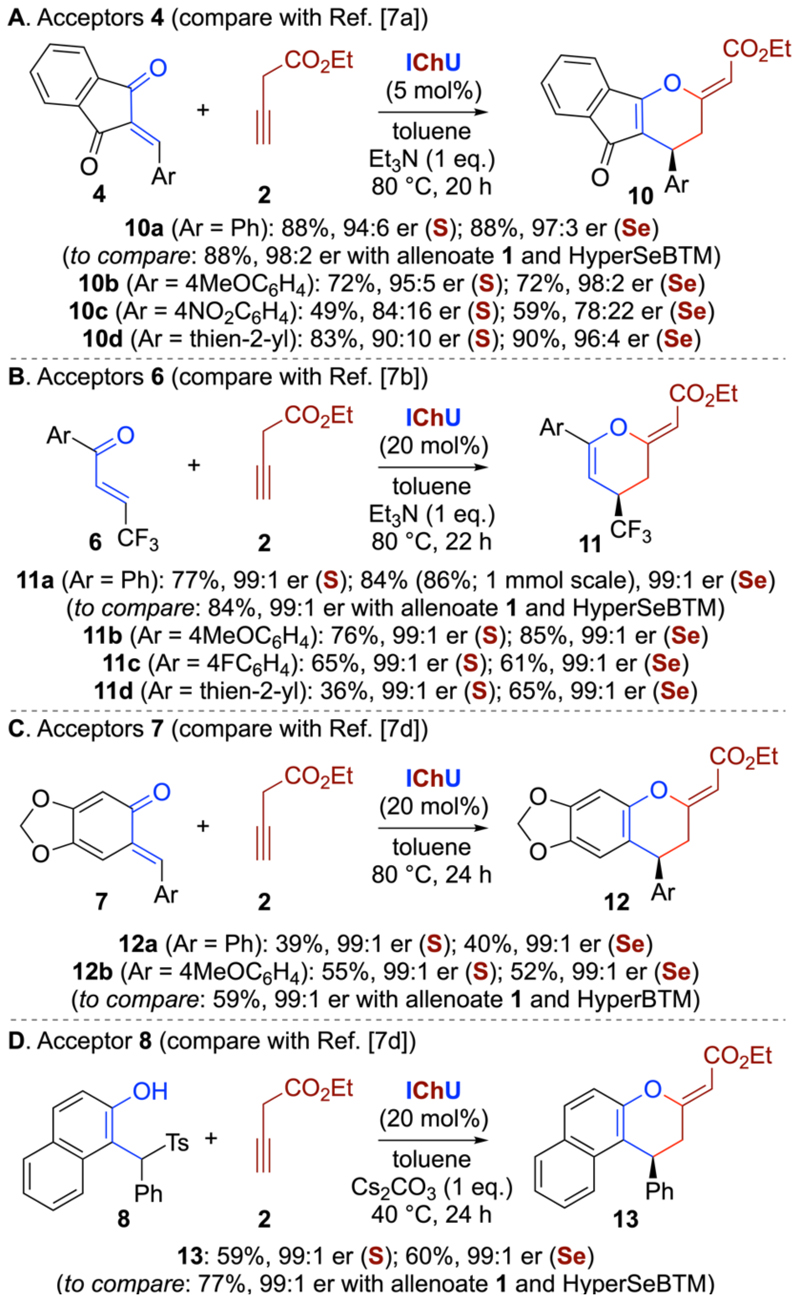
Asymmetric (4+2)-cycloadditions of alkyne 2 with various acceptors.

**Table 1:** Optimization and application scope of the (4+2)-cycloaddition of alkyne 2 with barbiturate-based acceptors 3

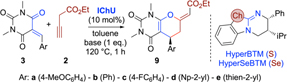							
Entry	Ar	2 (eq.)	IChU	base	Conv.^a^	Yield^b^	er^c^
1	**a**	3	S	K_2_CO_3_	quant.	75	80:20
2	**a**	3	Se	K_2_CO_3_	quant.	83	86:14
3	**a**	2.5	Se	K_2_CO_3_	60 %	36	86:14
4	**a**	2.5	Se	Cs_2_CO_3_	quant.	61	86:14
5	**a**	2.5	Se	Et_3_N	quant.	87	87:13
6	**a**	1.5	Se	Et_3_N	quant.	95 (96)^e^	87:13 (86:14)^d^
7	**a**	1.5	S	Et_3_N	quant.	87	80:20
8	**b**	1.5	Se	Et_3_N	quant.	84	79:21
9	**b**	1.5	S	Et_3_N	quant.	75	74:26
10	**c**	1.5	Se	Et_3_N	quant.	73	79:21
11	**c**	1.5	S	Et_3_N	quant.	64	71:29
12	**d**	1.5	Se	Et_3_N	quant.	87	72:28
13	**d**	1.5	S	Et_3_N	quant.	84	68:32
14	**e**	1.5	Se	Et_3_N	quant.	89 (86)^d^	84:16 (78:22)^d^
15	**e**	1.5	S	Et_3_N	quant.	81	76:24

a) Based on 3 (determined by ^1^H NMR of the crude product).

b) Isolated yields.

c) Determined by HPLC using a chiral stationary phase. The absolute configuration of the major enantiomer was assigned by comparison with our recent results^7b^

d) For comparison (given in brackets): Selected results for the formation of these products starting from allenoates 1 under the thereby optimized conditions7b.
