# Supplementary material for: Asymmetric Isochalcogenourea-Catalyzed Synthesis of 3,4-Dihydropyrans via (4+2)-Cycloadditions of Ethyl But-3-ynoate with Michael Acceptors
Source: Synlett. Author manuscript; Available in PMC 2025 Dec 1. (PMC7618023; doi:10.1055/a-2659-8340)
Supplement: Supporting Info [file EMS207327-supplement-Supporting_Info.pdf]

Asymmetric Isochalcogenourea-Catalyzed Synthesis of 3,4-Dihydropyrans via (4+2)-Cycloadditions of Ethyl But-3-ynoate with Michael Acceptors

Mario Hofer[a], Magdalena Piringer[a], Anna Scheucher[a], Lukas S. Vogl[a], Mario Waser[a]*

[a]Institute of Organic Chemistry, Johannes Kepler University Linz,
Altenberger Straße 69, 4040 Linz, Austria

Table of Contents

1 General information 1

2 Synthesis of ethyl but-3-ynoate (2) 2

3 IChU-catalyzed (4+2) annulation of ethyl but-2-ynoate (2) with Michael acceptors 3

3.1 General remarks 3

3.2 General procedure for the (4+2) annulation 3

4 Product characterization 4

5 Bibliography 10

6 NMR spectra of compounds 2 and 12a 11

7 EI-QMS spectrum of ethyl but-3-ynoate (2) 13

8 HPLC chromatograms of annulation products 9 – 13 14

1 General information

NMR spectra were recorded on a Bruker Avance III 300 MHz spectrometer with a broad band observe probe and a sample changer for 16 samples or a Bruker Avance DRX 500 MHz spectrometer which are all property of the Austro Czech NMR Research Center "RERI uasb". All NMR spectra were calibrated to the solvent residual peak (CDCl3: δ 7.26 ppm for 1H NMR and δ 77.16 ppm for 13C NMR). NMR data are reported as follows: chemical shift (δ / ppm), multiplicity (s = singlet, d = doublet, t = triplet, q = quartet, m = multiplet, bs = broad singlet), coupling constants (Hz), and integrals. High resolution mass spectra were obtained using an Agilent QTOF 6520 with ESI source. Preparative column chromatography was carried out using Davisil LC 60A 70– 200 MICRON silica gel. (Preparative) thin layer chromatography was performed on Macherey-Nagel pre-coated TLC plates (silica gel, 60 F254, 0.20 mm, ALUGRAM® Xtra SIL, respectively, 0.50 mm, SIL G-50). TLC plates were visualized under 254 nm UV lamp. Semipreparative HPLC was carried out using a Dionex Ultimate 3000 system with variable wavelength detection and a Grace Alltima Silica 10 µm 250x10 mm column. Enantiomeric ratios (e.r.) were determined by HPLC analysis using a Dionex Ultimate 3000 or a Shimadzu Prominence HPLC system with a CHIRAL ART Amylose-SA (4.6 mm × 250 mm, 5 µm), a CHIRAL ART Cellulose-SB (4.6 mm × 250 mm, 5 µm), a CHIRALCEL® OD-H (4.6 mm × 250 mm, 5 µm) or a CHIRALPAK® AD-H (4.6 mm × 250 mm, 5 µm) chiral stationary phase.

All chemicals were purchased from commercial suppliers and used without further purification unless otherwise stated. Technical grade solvents for extraction or chromatography (EtOAc, heptanes and DCM) were distilled prior to use.

HyperBTM was purchased from commercial sources. Its selenium analogue **HyperSeBTM**[1], barbiturate (**3**)[2], indandione (**4**)[3], trifluoromethyl enone (**6**)[4], and *o*-quinone methide (**7** and **8**)[5] based Michael acceptors were prepared according to literature.

2 Synthesis of ethyl but-3-ynoate (2)

The route for the synthesis of alkyne **X** is summarized in Scheme S1.

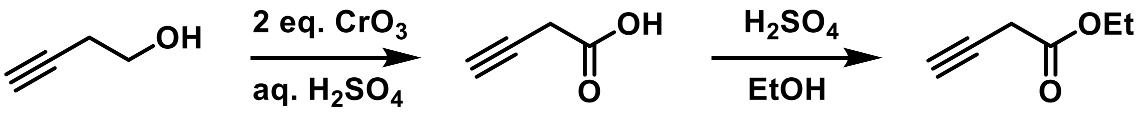

Scheme S1. Summarized reaction scheme for the synthesis of alkyne **X**.

The oxidation of but-3-yn-1-ol (**S-1**) to but-3-ynoic acid (**S-2**) was performed according to literature[6]. The esterification was done using a procedure based on literature[7]:

1.05 g (12.5 mmol) of but-3-ynoic acid (**S-2**) and 0.5 mL of H_2SO_4 were dissolved in 12 mL of EtOH. After stirring for 5 days at room temperature, 10 mL of H_2O were added. The solution was extracted four times with DCM, dried over Na_2SO_4 and concentrated under reduced pressure while cooled on an ice-water bath. Again, 10 mL of H_2O were added followed by extraction with DCM four times, drying over Na_2SO_4 and removing the solvent under reduced pressure while cooling the product on an ice-water bath. Ethyl but-3-ynoate (**2**) was obtained in 56 % yield (0.79 g, 7.0 mmol).

^{1}H NMR (300 MHz, $CDCl_3$, δ/ppm): 4.21 (q, J = 7.1 Hz, 2 H, O-CH_2), 3.28 (d, J = 2.8 Hz, 2 H, CH_2), 2.19 (t, J = 2.8 Hz, 1 H, ≡CH), 1.29 (t, J = 7.1 Hz, 3 H, CH_3); contains 6 % of allene **1**: 5.63 (t, J = 6.5 Hz, 1 H, =CH-), 5.21 (d, J = 6.5 Hz, 2 H, =CH_2), remaining CH_2 and CH_3 signals overlap with main product signals.

^{13}C NMR (75 MHz, $CDCl_3$, δ/ppm): 168.0 (C=O), 75.9 (≡C-), 71.8 (≡CH), 61.9 (OCH_2), 26.0 (CH_2), 14.2 (CH_3).

MS: EI-QMS (m/z): 112 (1 %, M), 67 (69 %, M – CH_3CH_2O), 39 (53 %, HC≡C-CH_2), 29 (100 %, CH_3CH_2). (please note that the compound is not detectable by ESI-TOF)

3 IChU-catalyzed (4+2) annulation of ethyl but-2-ynoate (2) with Michael acceptors

3.1 General remarks

Throughout the optimization process and the application scope, all products were obtained with high preference for the (Z)-diastereomer. The (E)-diastereomer was never identified in any of the crude NMR spectra. Possible trace amounts were removed chromatographically.

3.2 General procedure for the (4+2) annulation

Adapting procedures used in previous publications from our group[8–11], 17 µL of alkyne **2** (1.5 eq., 0.15 mmol), 100 µmol (1 eq.) of Michael acceptor, IChU-catalyst (**HyperBTM** or **HyperSeBTM**), and base were dissolved in 5 mL of toluene in a flame a flame-dried Schlenk flask under N_2 and stirred (exact reaction conditions summarized in Table S1). After the reaction was complete, the solution was filtered through a Na_2SO_4 plug and the solvent was evaporated under reduced pressure. After drying *in vacuo*, the crude product was purified using preparative TLC.

Table S1. Reaction conditions used for the application scope of the IChU-catalyzed (4+2) annulation.

Entry	Acceptor	Product	Time / h	Temperature / °C	Cat. loading / %	Base (1 eq.)
1	**3**	**9**	1	120	10	Et_3N
2	**4**	**10**	20	80	5	Et_3N
3	**6**	**11**	22	80	20	Et_3N
4	**7**	**12**	24	80	20	None[1]
5	**8**	**13**	24	40	20	Cs_2CO_3 [2]

[1]Similar results with and without base. [2]Cs_2CO_3 needed for *in situ* generation of quinone methide from precursor **8**. No additional base required for isomerization of alkyne **2**.

4 Product characterization

Ethyl (*S*,*Z*)-2-(5-(4-methoxyphenyl)-1,3-dimethyl-2,4-dioxo-1,2,3,4,5,6-hexahydro-7*H*-pyrano[2,3-*d*]pyrimidin-7-ylidene)acetate (9a)

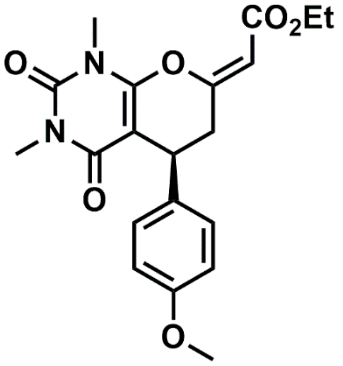

Product **9a** was synthesized according to the general procedure (Table S1, entry 1, scaled down by a factor of 2). The compound was obtained as an off-white viscous residue in 87 % (16.8 mg) and 80:20 *e.r.* and in 95 % (18.4 mg) yield and 87:13 *e.r.* when using **HyperBTM** or **HyperSeBTM**, respectively. The spectral data matched those found in literature[9].

HPLC (YMC-SB, *n*-hexane:*i*PrOH = 1:1, flow = 1.0 mL min^{-1}, T = 10 °C, λ = 210 nm): t_r: 19.5 min (major), 37.9 min (minor).

Ethyl (*S*,*Z*)-2-(1,3-dimethyl-2,4-dioxo-5-phenyl-1,2,3,4,5,6-hexahydro-7*H*-pyrano[2,3-*d*] pyrimidin-7-ylidene)acetate (9b)

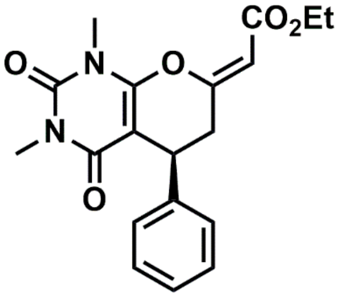

Product **9b** was synthesized according to the general procedure (Table S1, entry 1, scaled down by a factor of 2). The compound was obtained as an off-white viscous residue in 75 % (13.5 mg) and 74:26 *e.r.* and in 84 % (14.9 mg) yield and 79:21 *e.r.* when using **HyperBTM** or **HyperSeBTM**, respectively. The spectral data matched those found in literature[9].

HPLC (YMC-SB, *n*-hexane:*i*PrOH = 1:1, flow = 1.0 mL min^{-1}, T = 10 °C, λ = 210 nm): t_r: 14.4 min (major), 30.0 min (minor).

Ethyl (*S*,*Z*)-2-(5-(4-fluorophenyl)-1,3-dimethyl-2,4-dioxo-1,2,3,4,5,6-hexahydro-7*H*-pyrano [2,3-*d*]pyrimidin-7-ylidene)acetate (9c)

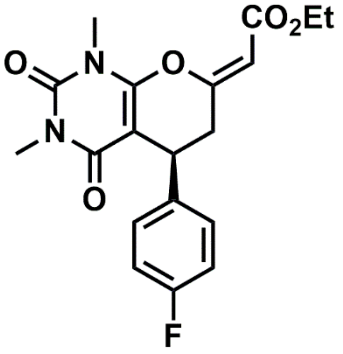

Product **9c** was synthesized according to the general procedure (Table S1, entry 1, scaled down by a factor of 2). The compound was obtained as an off-white viscous residue in 64 % (11.9 mg) and 71:29 *e.r.* and in 73 % (13.7 mg) yield and 79:21 *e.r.* when using **HyperBTM** or **HyperSeBTM**, respectively. The spectral data matched those found in literature[9].

HPLC (YMC-SB, *n*-hexane:*i*PrOH = 1:1, flow = 1.0 mL min^{-1}, T = 10 °C, λ = 210 nm): t_r: 13.4 min (major), 33.3 min (minor).

Ethyl (*S*,*Z*)-2-(1,3-dimethyl-5-(naphthalen-2-yl)-2,4-dioxo-1,2,3,4,5,6-hexahydro-7*H*-pyrano[2,3-*d*]pyrimidin-7-ylidene)acetate (9d)

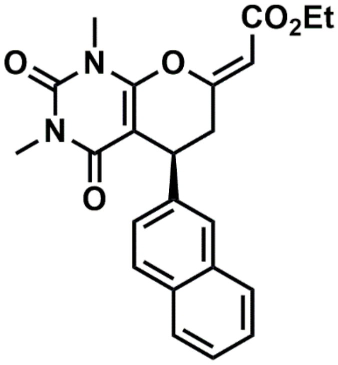

Product **9d** was synthesized according to the general procedure (Table S1, entry 1, scaled down by a factor of 2). The compound was obtained as an off-white viscous residue in 84 % (17.2 mg) and 68:32 *e.r.* and in 87 % (17.5 mg) yield and 72:28 *e.r.* when using **HyperBTM** or **HyperSeBTM**, respectively. The spectral data matched those found in literature[9].

HPLC (YMC-SB, *n*-hexane:*i*PrOH = 1:1, flow = 1.0 mL min^{-1}, T = 10 °C, λ = 210 nm): t_r: 18.9 min (major), 39.3 min (minor).

Ethyl (*R*,*Z*)-2-(1,3-dimethyl-2,4-dioxo-5-(thiophen-2-yl)-1,2,3,4,5,6-hexahydro-7*H*-pyrano[2,3-*d*]pyrimidin-7-ylidene)acetate (9e)

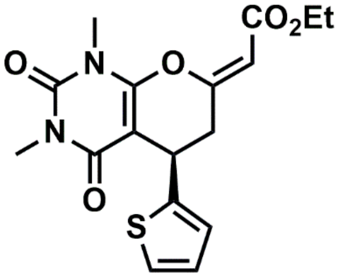

Product **9e** was synthesized according to the general procedure (Table S1, entry 1, scaled down by a factor of 2). The compound was obtained as an off-white viscous residue in 81 % (14.7 mg) and 76:24 *e.r.* and in 89 % (16.2 mg) yield and 84:16 *e.r.* when using **HyperBTM** or **HyperSeBTM**, respectively. The spectral data matched those found in literature[9].

HPLC (YMC-SB, *n*-hexane:*i*PrOH = 1:1, flow = 1.0 mL min^{-1}, T = 10 °C, λ = 210 nm): t_r: 18.6 min (major), 33.0 min (minor).

Ethyl (*S*,*Z*)-2-(4-(4-methoxyphenyl)-5-oxo-4,5-dihydroindeno[1,2-*b*]pyran-2(3*H*)-ylidene)acetate (10a)

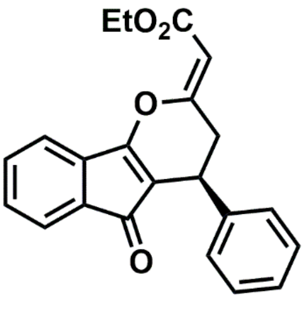

Product **10a** was synthesized according to the general procedure (Table S1, entry 2). The compound was obtained as a yellow waxy solid in 88 % (31.0 mg) and 94:6 *e.r.* and in 88 % (30.9 mg) yield and 97:3 *e.r.* when using **HyperBTM** or **HyperSeBTM**, respectively. The spectral data matched those found in literature[10].

HPLC (YMC-SA, *n*-hexane:*i*PrOH = 10:1, flow = 0.5 mL min^{-1}, T = 10 °C, λ = 220 nm): t_r: 21.7 min (minor), 30.6 min (major).

Ethyl (*S*,*Z*)-2-(4-(4-methoxyphenyl)-5-oxo-4,5-dihydroindeno[1,2-*b*]pyran-2(3*H*)-ylidene) acetate (10b)

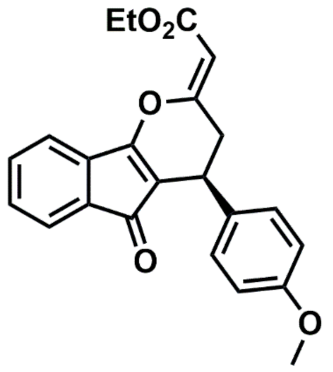

Product **10b** was synthesized according to the general procedure (Table S1, entry 2). The compound was obtained as a yellow waxy solid in 72 % (27.0 mg) and 95:5 *e.r.* and in 72 % (26.9 mg) yield and 98:2 *e.r.* when using **HyperBTM** or **HyperSeBTM**, respectively. The spectral data matched those found in literature[10].

HPLC (YMC-SA, *n*-hexane:*i*PrOH = 4:1, flow = 0.5 mL min^{-1}, T = 10 °C, λ = 220 nm): t_r: 22.1 min (minor), 30.9 min (major).

Ethyl (*S*,*Z*)-2-(4-(4-nitrophenyl)-5-oxo-4,5-dihydroindeno[1,2-*b*]pyran-2(3*H*)-ylidene)acetate (10c)

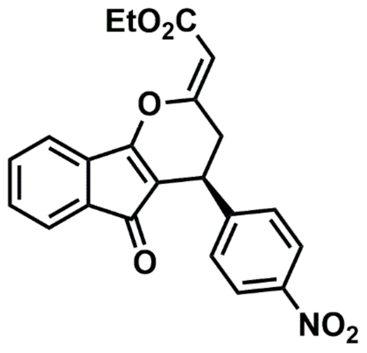

Product **10c** was synthesized according to the general procedure (Table S1, entry 2). The compound was obtained as a yellow waxy solid in 49 % (19.5 mg) and 84:16 *e.r.* and in 59 % (23.2 mg) yield and 78:22 *e.r.* when using **HyperBTM** or **HyperSeBTM**, respectively. The spectral data matched those found in literature[10].

HPLC (YMC-SA, *n*-hexane:*i*PrOH = 4:1, flow = 1.0 mL min^{-1}, T = 10 °C, λ = 220 nm): t_r: 34.9 min (minor), 53.8 min (major).

Ethyl (*R*,*Z*)-2-(5-oxo-4-(thiophen-2-yl)-4,5-dihydroindeno[1,2-*b*]pyran-2(3*H*)-ylidene)acetate (10d)

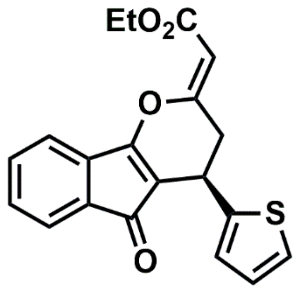

Product **10d** was synthesized according to the general procedure (Table S1, entry 2). The compound was obtained as a yellow waxy solid in 83 % (29.0 mg) and 90:10 *e.r.* and in 90 % (31.6 mg) yield and 96:4 *e.r.* when using **HyperBTM** or **HyperSeBTM**, respectively. The spectral data matched those found in literature[10].

HPLC (YMC-SA, *n*-hexane:*i*PrOH = 4:1, flow = 0.5 mL min^{-1}, T = 10 °C, λ = 220 nm): t_r: 18.2 min (minor), 21.9 min (major).

Ethyl (*R*,*Z*)-2-(6-phenyl-4-(trifluoromethyl)-3,4-dihydro-2*H*-pyran-2-ylidene)acetate (11a)

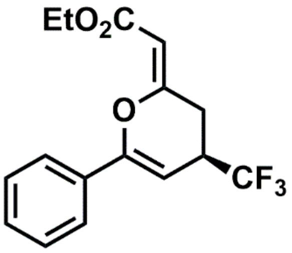

Product **11a** was synthesized according to the general procedure (Table S1, entry 3). The compound was obtained as an off-white solid in 77 % (24.2 mg) and >99:1 *e.r.* and in 84 % (26.1 mg) yield and >99:1 *e.r.* when using **HyperBTM** or **HyperSeBTM**, respectively. The spectral data matched those found in literature[9].

HPLC (CHIRALPAK AD-H, *n*-hexane:*i*PrOH = 9:1, flow = 1.0 mL min^{-1}, T = 20 °C, λ = 254 nm): t_r: 4.9 min (major), 6.3 min (minor).

Ethyl (*R*,*Z*)-2-(6-(4-methoxyphenyl)-4-(trifluoromethyl)-3,4-dihydro-2*H*-pyran-2-ylidene) acetate (11b)

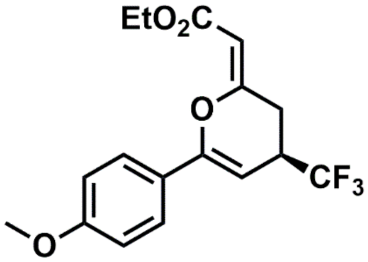

Product **11b** was synthesized according to the general procedure (Table S1, entry 3). The compound was obtained as an off-white solid in 76 % (26.1 mg) and >99:1 *e.r.* and in 85 % (29.1 mg) yield and >99:1 *e.r.* when using **HyperBTM** or **HyperSeBTM**, respectively. The spectral data matched those found in literature[9].

HPLC (CHIRALPAK OD-H, *n*-hexane:*i*PrOH = 4:1, flow = 0.5 mL min^{-1}, T = 20 °C, λ = 254 nm): t_r: 16.3 min (minor), 17.8 min (major).

Ethyl (*R*,*Z*)-2-(6-(4-fluorophenyl)-4-(trifluoromethyl)-3,4-dihydro-2*H*-pyran-2-ylidene)acetate (11c)

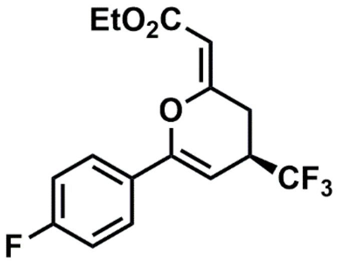

Product **11c** was synthesized according to the general procedure (Table S1, entry 3). The compound was obtained as an off-white solid in 65 % (21.7 mg) and >99:1 *e.r.* and in 61 % (20.1 mg) yield and >99:1 *e.r.* when using **HyperBTM** or **HyperSeBTM**, respectively. The spectral data matched those found in literature[9].

HPLC (CHIRALPAK OD-H, *n*-hexane:*i*PrOH = 4:1, flow = 0.5 mL min^{-1}, T = 20 °C, λ = 254 nm): t_r: 9.9 min (minor), 12.5 min (major).

Ethyl (*R*,*Z*)-2-(6-(thiophen-2-yl)-4-(trifluoromethyl)-3,4-dihydro-2*H*-pyran-2-ylidene)acetate (11d)

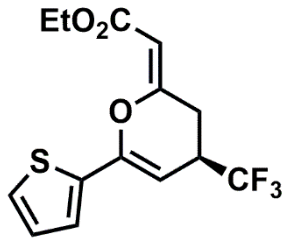

Product **11d** was synthesized according to the general procedure (Table S1, entry 3). The compound was obtained as an off-white solid in 36 % (11.4 mg) and >99:1 *e.r.* and in 65 % (20.8 mg) yield and >99:1 *e.r.* when using **HyperBTM** or **HyperSeBTM**, respectively. The spectral data matched those found in literature[9].

HPLC (CHIRALPAK OD-H, *n*-hexane:*i*PrOH = 4:1, flow = 0.5 mL min^{-1}, T = 20 °C, λ = 254 nm): t_r: 11.6 min (minor), 12.2 min (major).

Ethyl (*S*,*Z*)-2-(8-phenyl-7,8-dihydro-6*H*-[1,3]dioxolo[4,5-*g*]chromen-6-ylidene)acetate (12a)

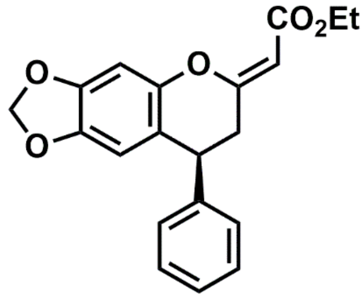

Product **12a** was synthesized according to the general procedure (Table S1, entry 4). The compound was obtained as a yellow oil in 39 % (13.2 mg) and 99:1 *e.r.* and in 40 % (13.5 mg) yield and 99:1 *e.r.* when using **HyperBTM** or **HyperSeBTM**, respectively.

^{1}H-NMR (300 MHz, $CDCl_3$, 298 K) δ / ppm: 7.35 – 7.22 (m, 3H, Ar-**H**), 7.14 – 7.10 (m, 2H, Ar-**H**), 6.74 (s, 1H, Ar-**H**), 6.30 (s, 1H, Ar-**H**), 5.91 – 5.90 (m, 2H, -C**H₂**), 4.92 (s, 1H, -C**H**), 4.17 (qd, J = 7.2, 1.2 Hz, 2H, -C**H₂**), 4.04 (dd, J = 7.5, 5.3 Hz, 1H, -C**H**), 2.88 (ddd, J = 14.7, 5.3, 1.0 Hz, 1H, -C**H₂**), 2.73 (ddd, J = 14.7, 7.5, 0.9 Hz, 1H, -C**H₂**), 1.28 (t, J = 7.2 Hz, 3H, -C**H₃**).

^{13}C-NMR (75 MHz, $CDCl_3$, 298 K) δ / ppm: 164.8 (1 **C**), 161.6 (1 **C**), 147.4 (1 **C**), 146.2 (1 **C**), 143.4 (1 **C**), 141.7 (1 **C**), 128.9 (1 **C**), 127.9 (1 **C**), 127.3 (1 **C**), 117.6 (1 **C**), 107.6 (1 **C**), 101.4 (1 **C**), 99.1 (1 **C**), 96.8 (1 **C**), 59.6 (1 **C**), 40.1 (1 **C**), 36.4 (1 **C**), 14.4 (1 **C**).

HRMS (ESI-TOF): *m/z* calculated for $C_{20}H_{18}O_5$: 339.1227 $[M+H]^+$, found: 339.1229.

$[\alpha]_D^{20}$(c = 1, $CHCl_3$, e.r. 99:1): +12.9.

HPLC (CHIRALPAK® AD-H, *n*-hexane:*i*PrOH = 4:1, flow = 1.0 mL min^{-1}, T = 10 °C, λ = 250 nm): t_r: 10.9 min (minor), 14.5 min (major).

Ethyl (*S*,*Z*)-2-(8-(4-methoxyphenyl)-7,8-dihydro-6*H*-[1,3]dioxolo[4,5-*g*]chromen-6-ylidene) acetate (12b)

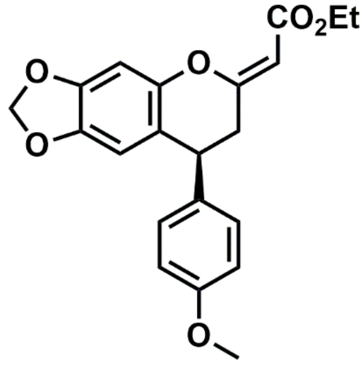

Product **12b** was synthesized according to the general procedure (Table S1, entry 4). The compound was obtained as a yellow oil in 55 % (20.3 mg) and 99:1 *e.r.* and in 52 % (19.2 mg) yield and 99:1 *e.r.* when using **HyperBTM** or **HyperSeBTM**, respectively. The spectral data matched those found in literature[11].

HPLC (YMC-SB, *n*-hexane:*i*PrOH = 4:1, flow = 1.0 mL min^{-1}, T = 10 °C, λ = 250 nm): t_r: 18.5 min (minor), 25.4 min (major).

Ethyl (*S*,*Z*)-2-(1-phenyl-1,2-dihydro-3*H*-benzo[*f*]chromen-3-ylidene)acetate (13)

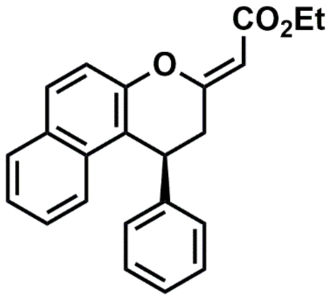

Product **13** was synthesized according to the general procedure (Table S1, entry 5). After additional purification via semi-preparative HPLC, the compound was obtained as a yellowish white amorphous residue in 59 % and 99:1 *e.r.* and in 60 % yield and 99:1 *e.r.* when using **HyperBTM** or **HyperSeBTM**, respectively. The spectral data matched those found in literature[11].

HPLC (YMC-SB, *n*-hexane:*i*PrOH = 4:1, flow = 1.0 mL min^{-1}, T = 10 °C, λ = 272 nm): t_r: 8.7 min (major), 24.3 min (minor).

5 Bibliography

(1) Young, C. M.; Elmi, A.; Pascoe, D. J.; Morris, R. K.; McLaughlin, C.; Woods, A. M.; Frost, A. B.; La Houpliere, A. de; Ling, K. B.; Smith, T. K. et al., *Angew. Chem. Int. Ed.* **2020**, *59*, 3705–3710.

(2) Liu, H.; Liu, Y.; Yuan, C.; Wang, G.-P.; Zhu, S.-F.; Wu, Y.; Wang, B.; Sun, Z.; Xiao, Y.; Zhou, Q.-L. et al., *Org. Lett.* **2016**, *18*, 1302–1305.

(3) Bano, B.; Kanwal; Khan, K. M.; Begum, F.; Lodhi, M. A.; Salar, U.; Khalil, R.; Ul-Haq, Z.; Perveen, S., *Bioorg. Chem.* **2018**, *81*, 658–671.

(4) Stark, D. G.; Morrill, L. C.; Yeh, P.-P.; Slawin, A. M. Z.; O'Riordan, T. J. C.; Smith, A. D., *Angew. Chem. Int. Ed.* **2013**, *52*, 11642–11646.

(5) (a) Meisinger, N.; Roiser, L.; Monkowius, U.; Himmelsbach, M.; Robiette, R.; Waser, M., *Chem. Eur. J.* **2017**, *23*, 5137–5142. (b) Gross, C.; Eitzinger, A.; Hampel, N.; Mayer, P.; Ofial, A. R., *Chem. Eur. J.* **2025**, *31*, e202403785.

(6) Kiattisewee, C.; Kaidad, A.; Jiarpinitnun, C.; Luanphaisarnnont, T., *Monatsh. Chem.* **2018**, *149*, 1059–1068.

(7) Snider, B. B.; Spindell, D. K., *J. Org. Chem.* **1980**, *45*, 5017–5020.

(8) Hofer, M.; Himmelsbach, M.; Monkowius, U.; Waser, M., *ChemCatChem* **2025**, *17*, e202500452.

(9) Piringer, M.; Hofer, M.; Vogl, L. S.; Mayer, P.; Waser, M., *Adv. Synth. Catal.* **2024**, *366*, 2115–2122.

(10) Vogl, L. S.; Mayer, P.; Robiette, R.; Waser, M., *Angew. Chem. Int. Ed.* **2024**, *63*, e202315345.

(11) Scheucher, A.; Gross, C.; Piringer, M.; Novacek, J.; Ofial, A. R.; Waser, M., *Org. Biomol. Chem.* **2025**, *23*, 827–834.

6 NMR spectra of compounds 2 and 12a

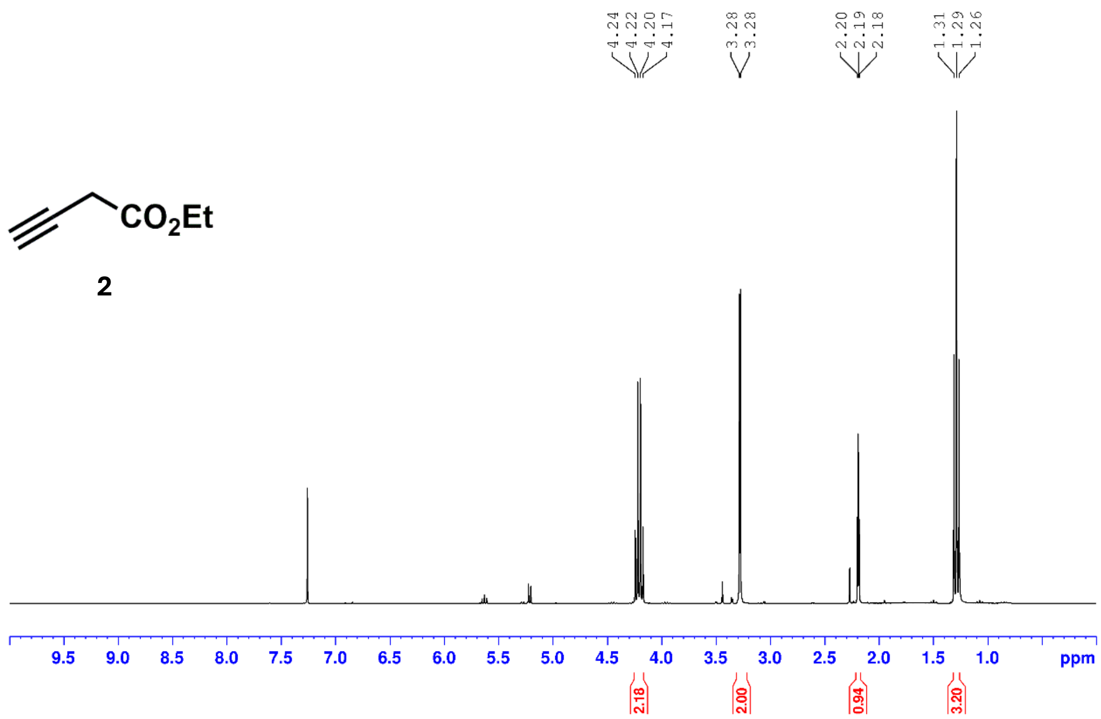

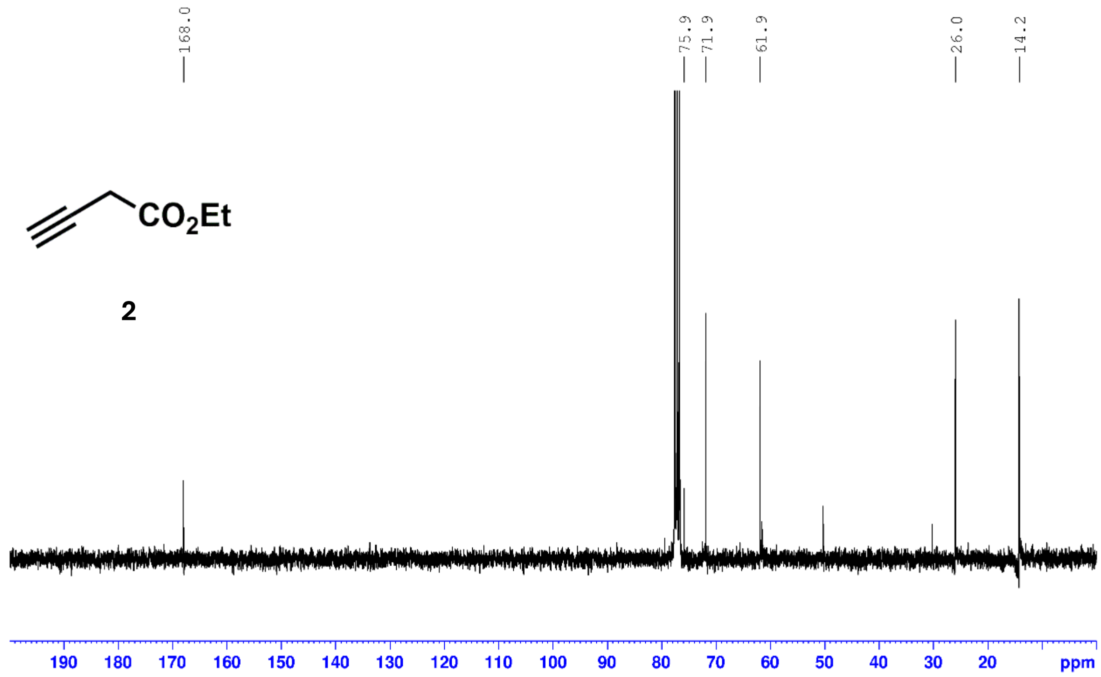

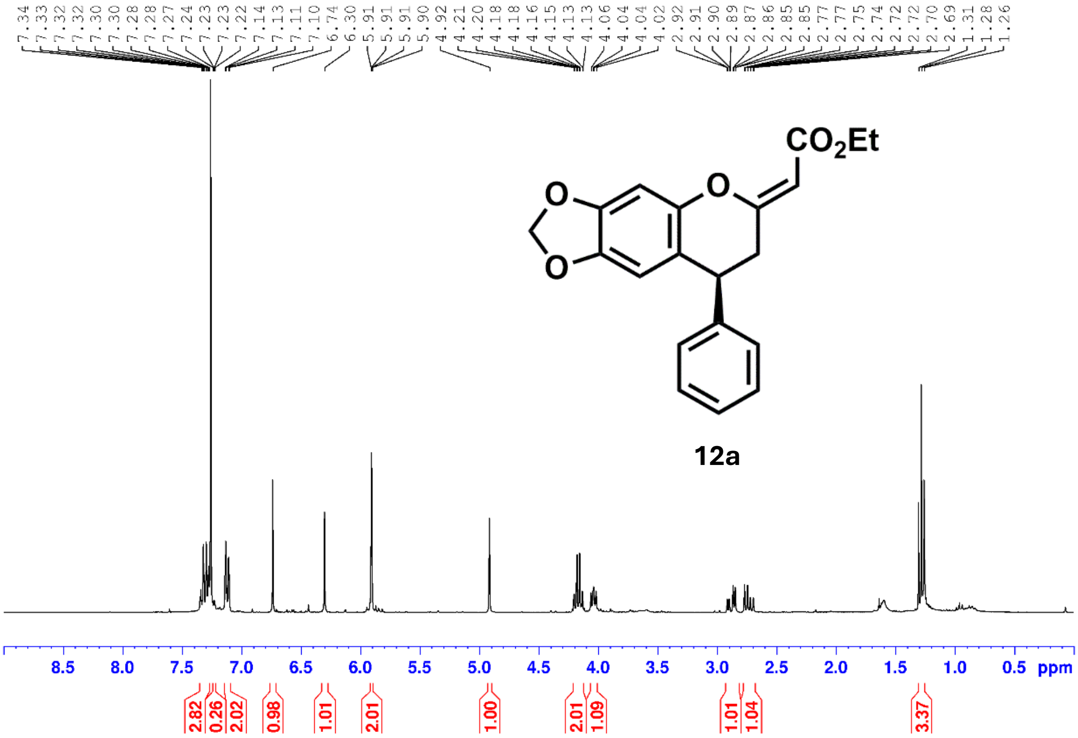
7.34
7.33
7.32
7.32
7.30
7.30
7.28
7.28
7.27
7.24
7.23
7.23
7.22
7.14
7.13
7.11
7.10
6.74
6.30
5.91
5.91
5.91
5.90
4.92
4.21
4.20
4.18
4.18
4.16
4.15
4.13
4.13
4.06
4.04
4.04
4.02
2.92
2.91
2.90
2.89
2.87
2.86
2.85
2.85
2.77
2.77
2.75
2.74
2.72
2.72
2.70
2.69
1.31
1.28
1.26
CO2Et
O
O
O
12a
8.5
8.0
7.5
7.0
6.5
6.0
5.5
5.0
4.5
4.0
3.5
3.0
2.5
2.0
1.5
1.0
0.5
ppm
2.82
0.26
2.02
0.98
1.01
2.01
1.00
2.01
1.09
1.01
1.04
3.37

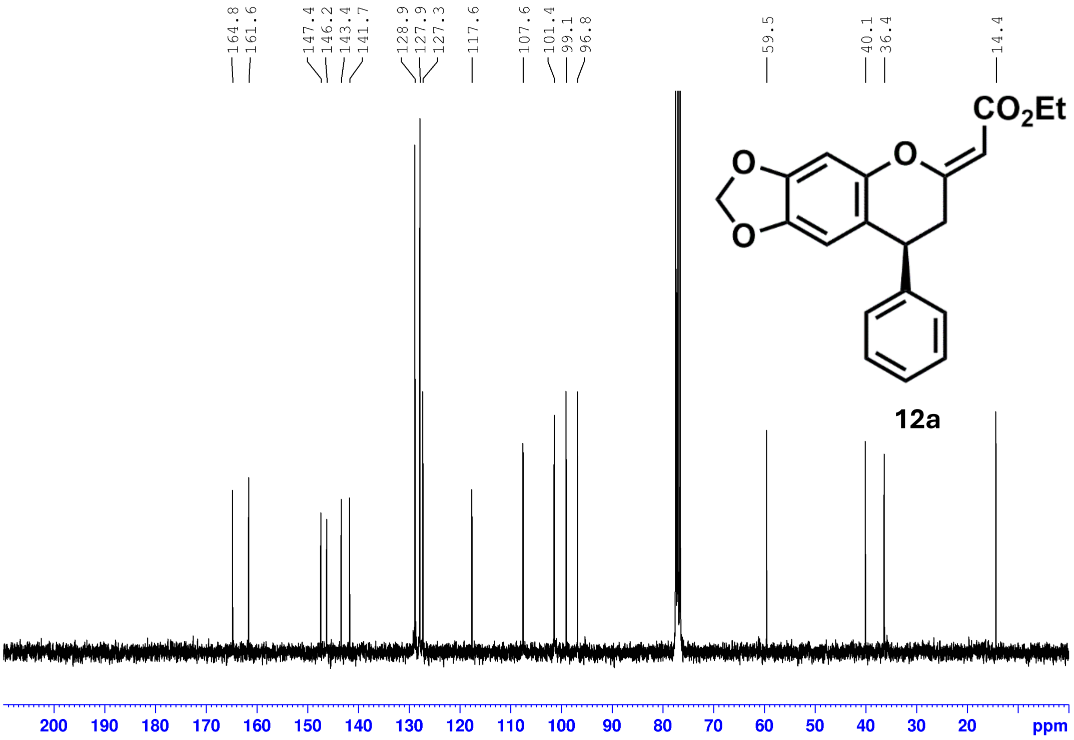
164.8
161.6
147.4
146.2
143.4
141.7
128.9
127.9
127.3
117.6
107.6
101.4
99.1
96.8
59.5
40.1
36.4
14.4
CO2Et
O
O
O
12a
200
190
180
170
160
150
140
130
120
110
100
90
80
70
60
50
40
30
20
ppm

7 EI-QMS spectrum of ethyl but-3-ynoate (2)

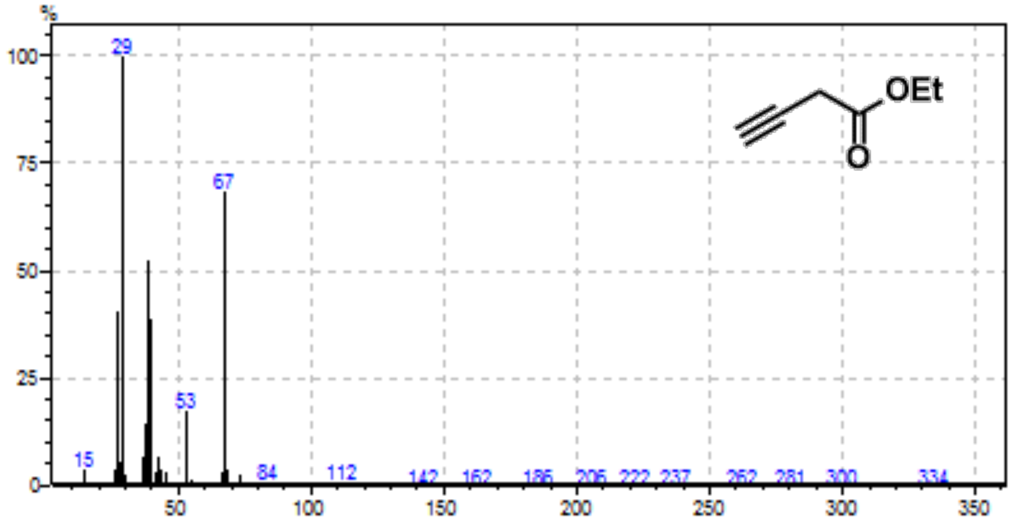

8 HPLC chromatograms of annulation products 9 – 13

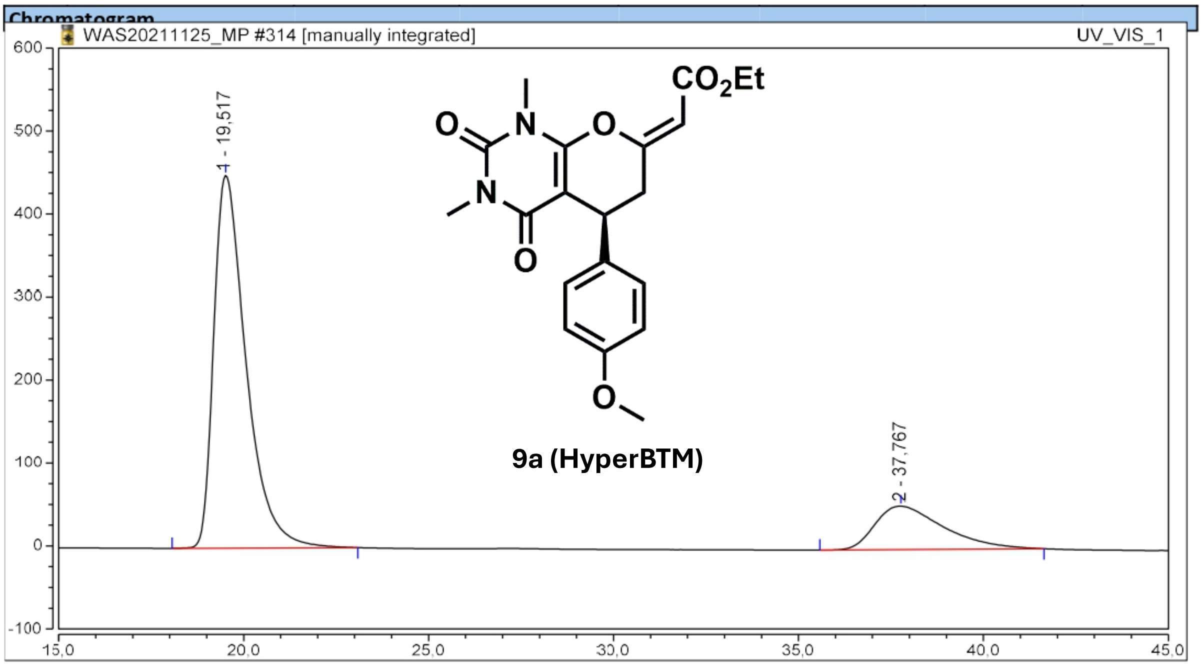

Integration Results							
No.	Peak Name	Retention Time min	Area mAU*min	Height mAU	Relative Area %	Relative Height %	Amount n.a.
1		19,517	454,521	449,364	80,13	89,55	n.a.
2		37,767	112,723	52,449	19,87	10,45	n.a.
**Total:**			**567,244**	**501,813**	**100,00**	**100,00**	

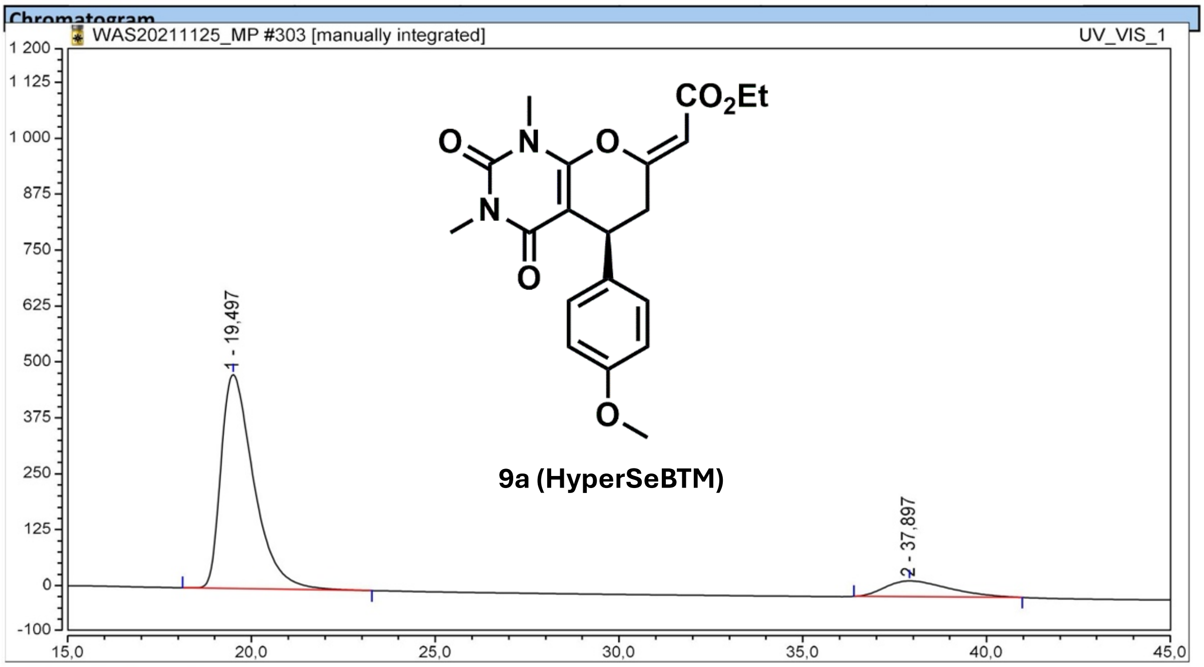

Integration Results							
No.	Peak Name	Retention Time min	Area mAU*min	Height mAU	Relative Area %	Relative Height %	Amount n.a.
1		19,497	494,882	478,111	87,34	93,12	n.a.
2		37,897	71,732	35,311	12,66	6,88	n.a.
**Total:**			**566,614**	**513,422**	**100,00**	**100,00**	

Chromatogram

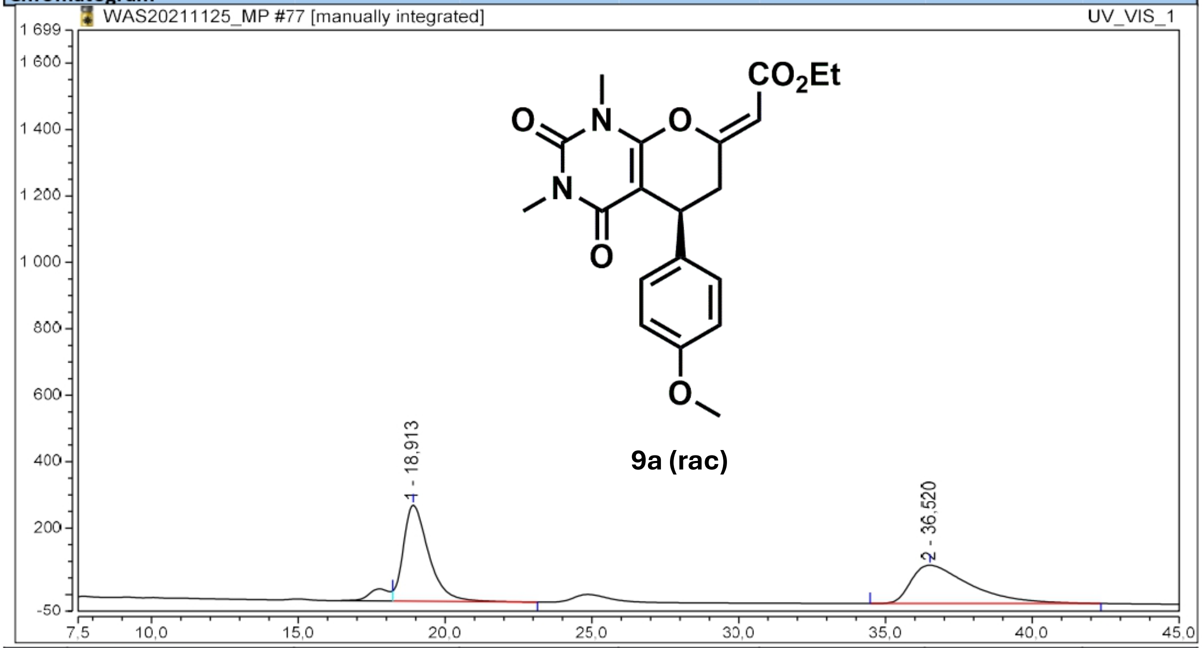

Integration Results

No.	Peak Name	Retention Time<br>min	Area<br>mAU*min	Height<br>mAU	Relative Area<br>%	Relative Height<br>%	Amount<br>n.a.
1		18,913	289,176	288,895	51,84	71,50	n.a.
2		36,520	268,604	115,181	48,16	28,50	n.a.
**Total:**			**557,779**	**404,076**	**100,00**	**100,00**	

Chromatogram

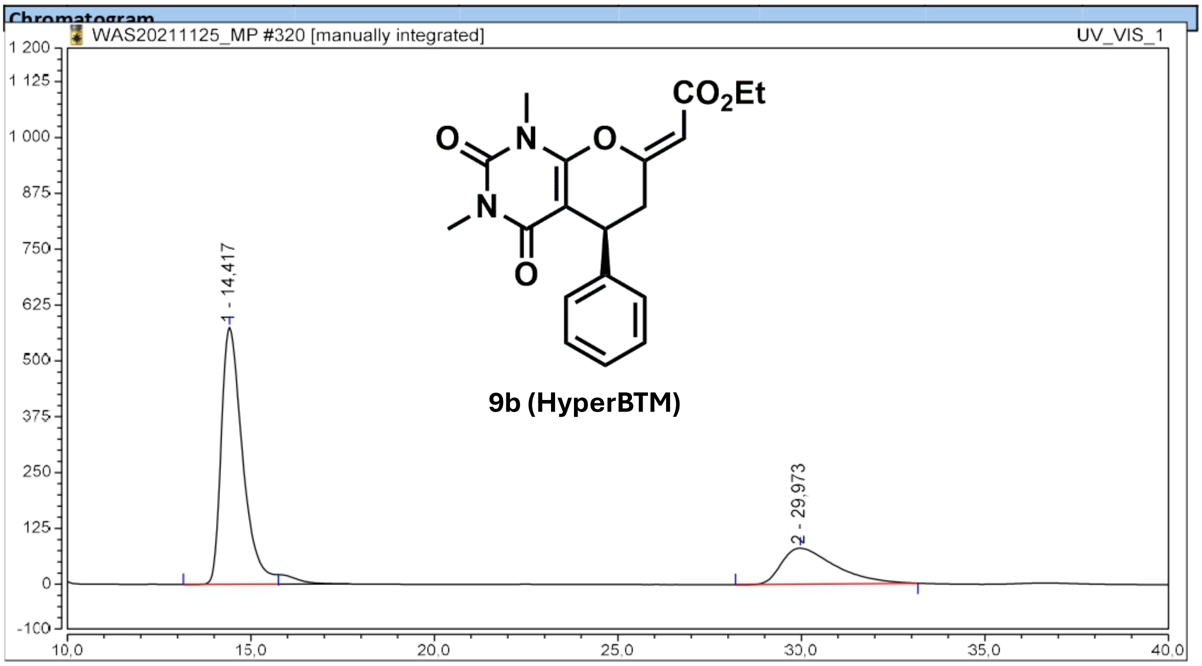

Integration Results

No.	Peak Name	Retention Time<br>min	Area<br>mAU*min	Height<br>mAU	Relative Area<br>%	Relative Height<br>%	Amount<br>n.a.
1		14,417	386,936	574,899	74,12	87,73	n.a.
2		29,973	135,114	80,411	25,88	12,27	n.a.
**Total:**			**522,050**	**655,310**	**100,00**	**100,00**	

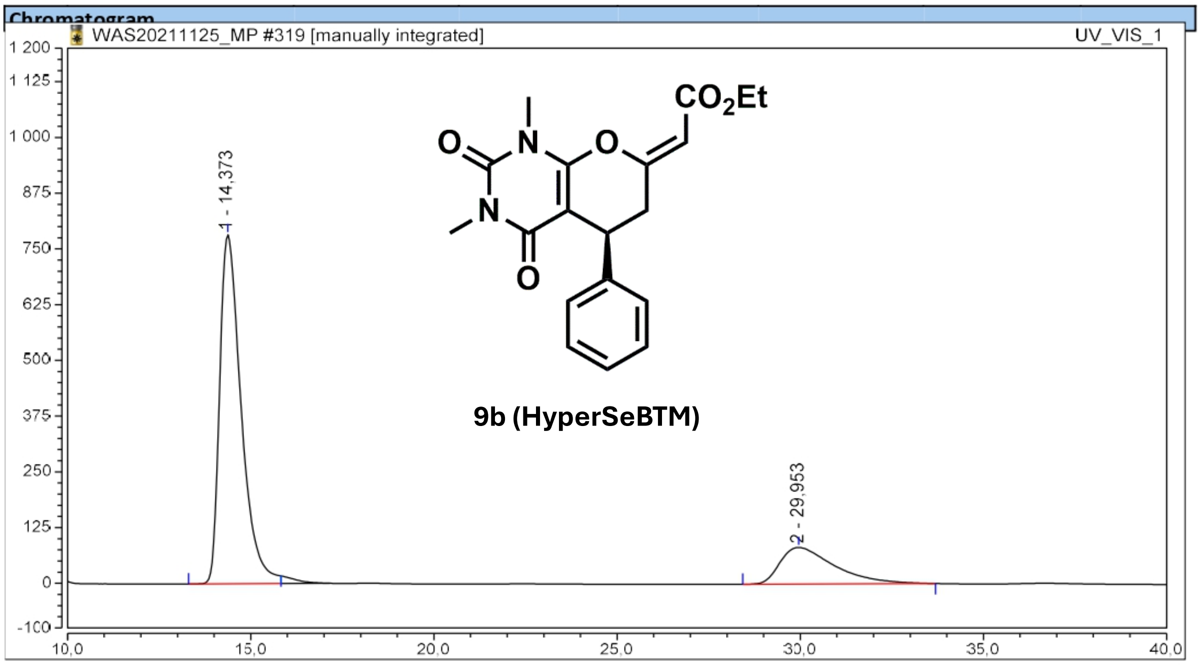

Integration Results							
No.	Peak Name	Retention Time min	Area mAU*min	Height mAU	Relative Area %	Relative Height %	Amount n.a.
1		14,373	530,741	782,557	78,97	90,50	n.a.
2		29,953	141,346	82,105	21,03	9,50	n.a.
**Total:**			**672,087**	**864,662**	**100,00**	**100,00**	

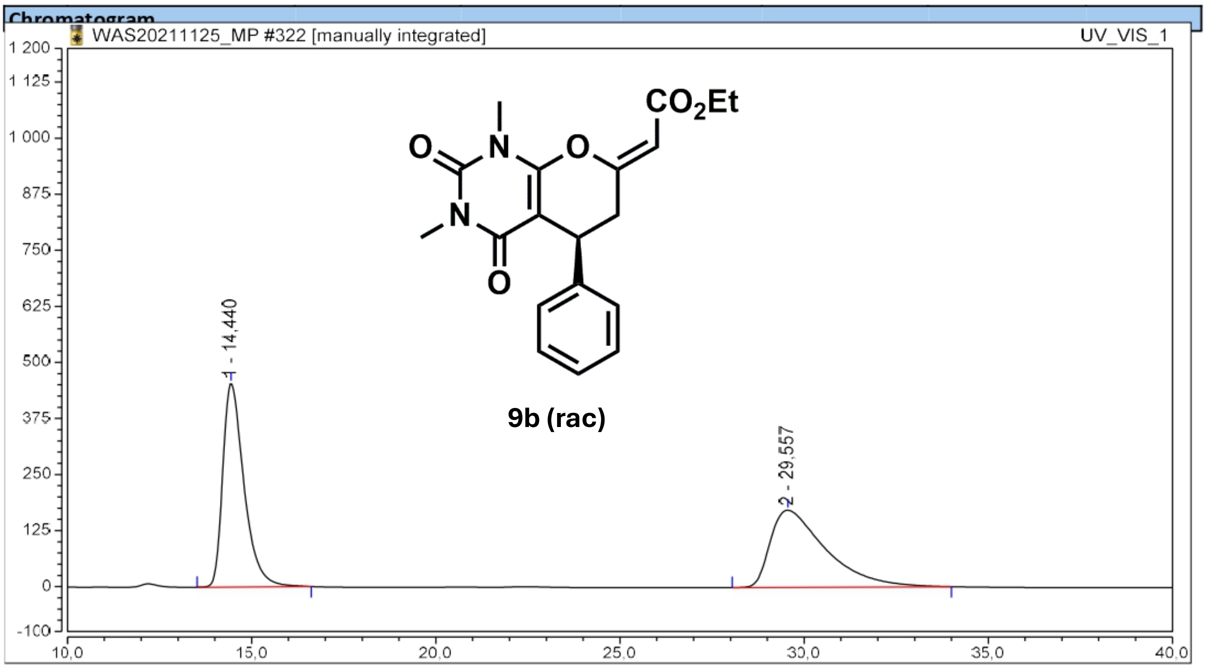

Integration Results							
No.	Peak Name	Retention Time min	Area mAU*min	Height mAU	Relative Area %	Relative Height %	Amount n.a.
1		14,440	304,305	454,257	50,32	72,52	n.a.
2		29,557	300,443	172,107	49,68	27,48	n.a.
**Total:**			**604,748**	**626,364**	**100,00**	**100,00**	

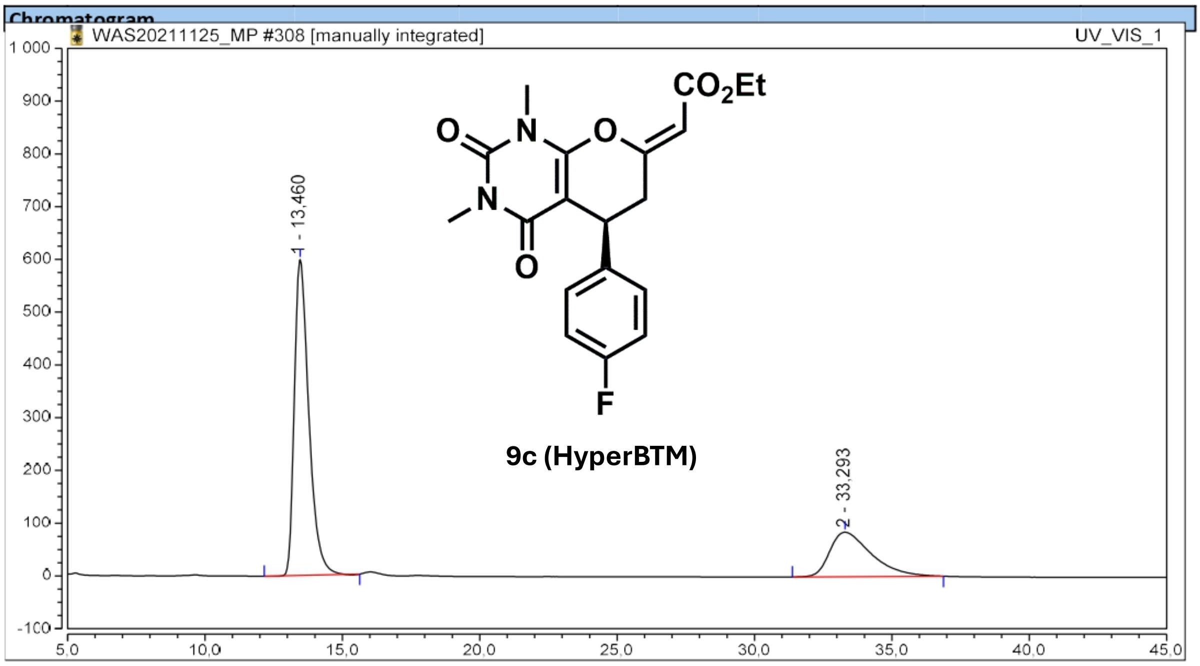

Integration Results							
No.	Peak Name	Retention Time min	Area mAU*min	Height mAU	Relative Area %	Relative Height %	Amount n.a.
1		13,460	364,441	598,776	71,08	87,63	n.a.
2		33,293	148,263	84,499	28,92	12,37	n.a.
**Total:**			**512,705**	**683,275**	**100,00**	**100,00**	

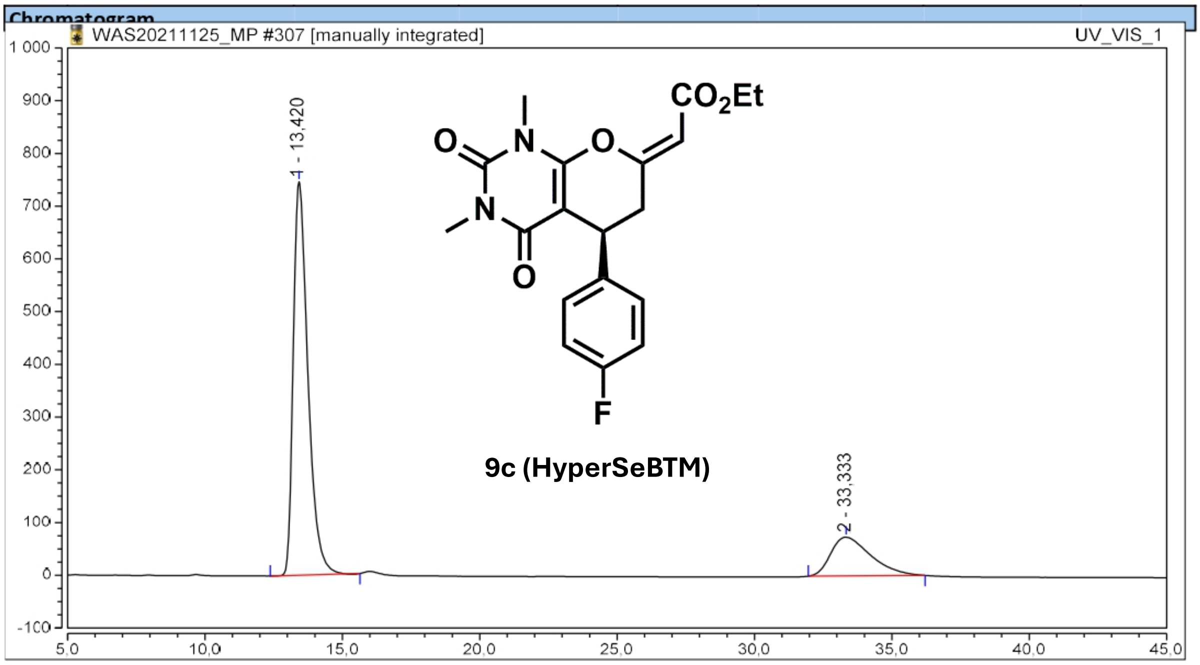

Integration Results							
No.	Peak Name	Retention Time min	Area mAU*min	Height mAU	Relative Area %	Relative Height %	Amount n.a.
1		13,420	459,517	746,309	78,74	91,04	n.a.
2		33,333	124,059	73,479	21,26	8,96	n.a.
**Total:**			**583,576**	**819,788**	**100,00**	**100,00**	

Chromatogram

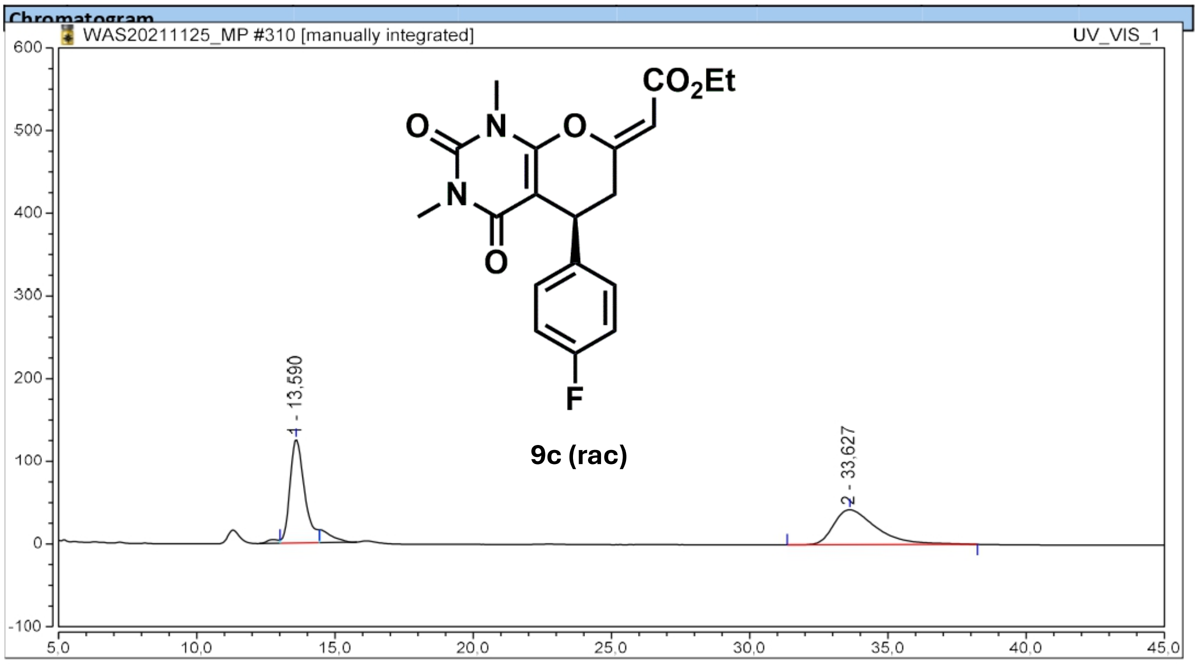

Integration Results

No.	Peak Name	Retention Time min	Area mAU*min	Height mAU	Relative Area %	Relative Height %	Amount n.a.
1		13,590	76,951	125,217	50,35	74,76	n.a.
2		33,627	75,873	42,285	49,65	25,24	n.a.
**Total:**			**152,824**	**167,502**	**100,00**	**100,00**	

Chromatogram

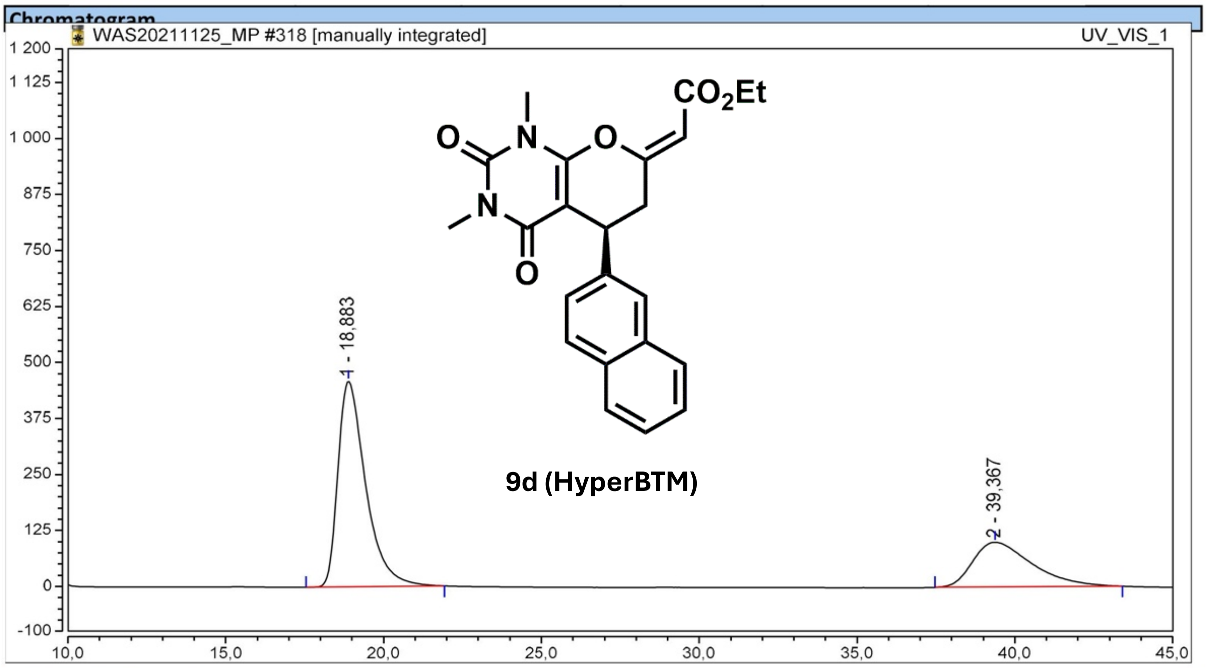

Integration Results

No.	Peak Name	Retention Time min	Area mAU*min	Height mAU	Relative Area %	Relative Height %	Amount n.a.
1		18,883	467,417	458,651	68,25	82,11	n.a.
2		39,367	217,474	99,947	31,75	17,89	n.a.
**Total:**			**684,891**	**558,598**	**100,00**	**100,00**	

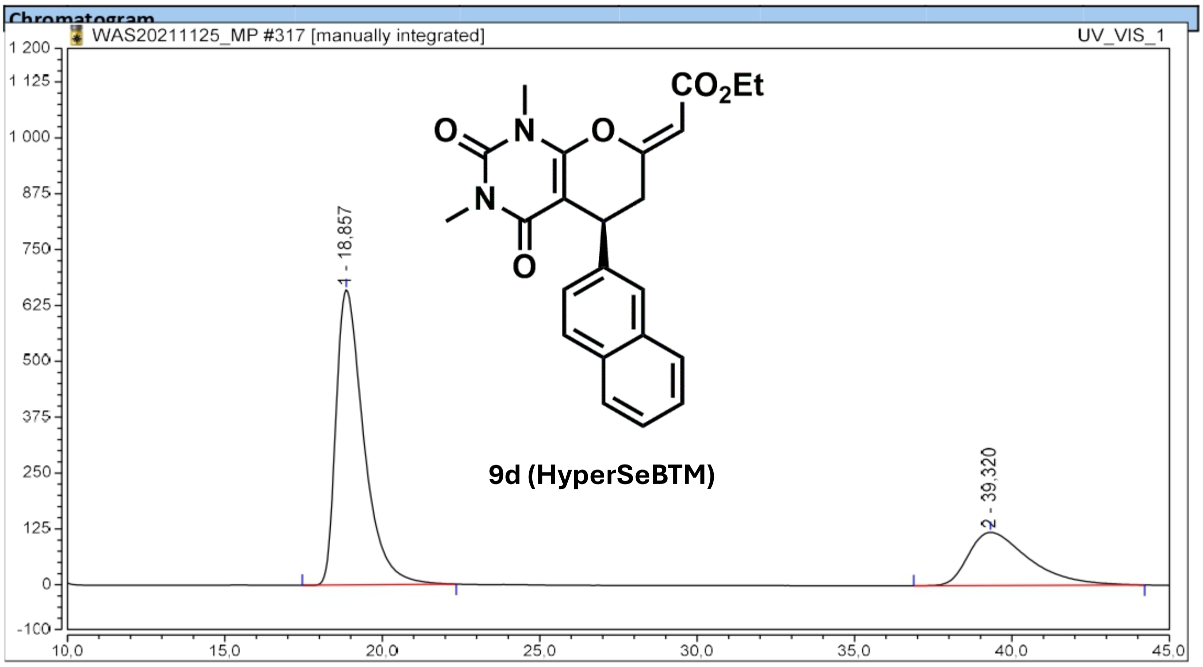

Integration Results

No.	Peak Name	Retention Time min	Area mAU*min	Height mAU	Relative Area %	Relative Height %	Amount n.a.
1		18,857	675,511	660,091	71,85	84,74	n.a.
2		39,320	264,652	118,894	28,15	15,26	n.a.
**Total:**			**940,163**	**778,985**	**100,00**	**100,00**	

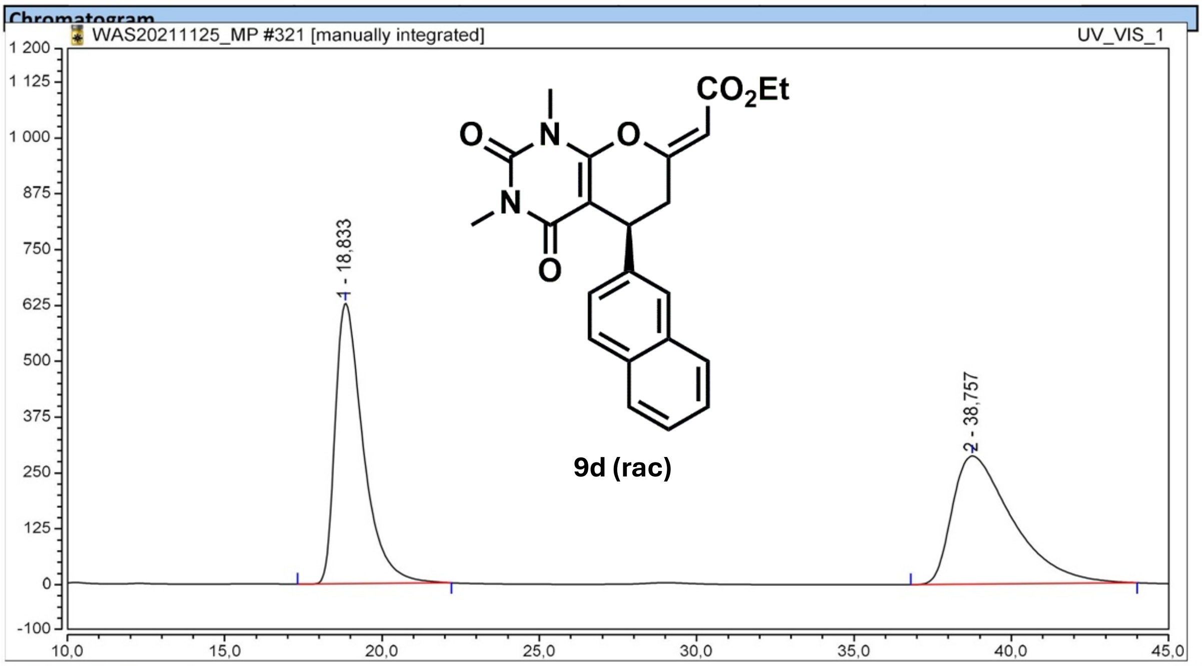

Integration Results

No.	Peak Name	Retention Time min	Area mAU*min	Height mAU	Relative Area %	Relative Height %	Amount n.a.
1		18,833	647,865	627,889	50,39	68,64	n.a.
2		38,757	637,864	286,917	49,61	31,36	n.a.
**Total:**			**1285,729**	**914,806**	**100,00**	**100,00**	

Chromatogram

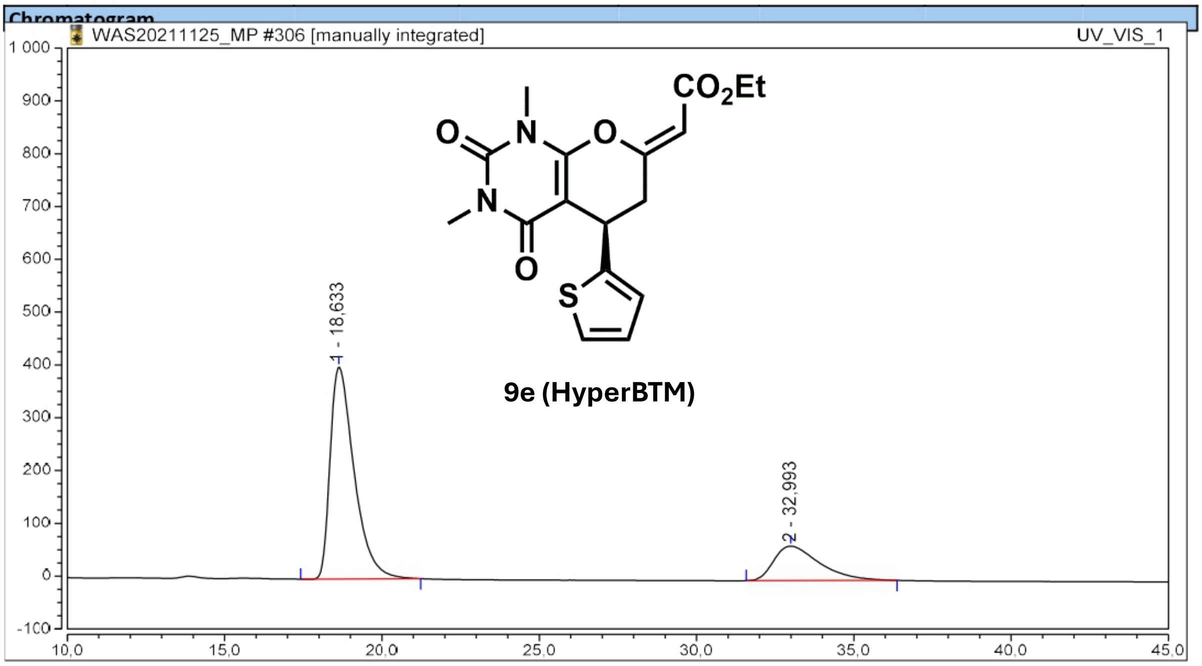

Integration Results							
No.	Peak Name	Retention Time<br>min	Area<br>mAU*min	Height<br>mAU	Relative Area<br>%	Relative Height<br>%	Amount<br>n.a.
1		18,633	354,751	401,750	76,38	86,03	n.a.
2		32,993	109,692	65,211	23,62	13,97	n.a.
**Total:**			**464,443**	**466,961**	**100,00**	**100,00**	

Chromatogram

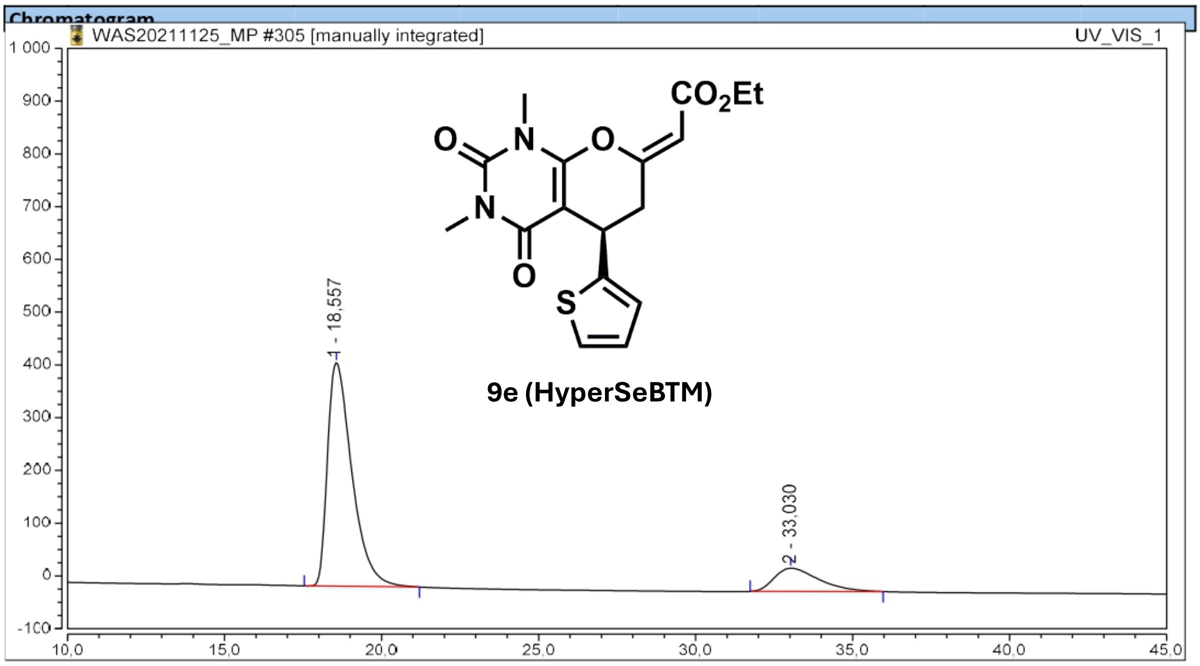

Integration Results							
No.	Peak Name	Retention Time<br>min	Area<br>mAU*min	Height<br>mAU	Relative Area<br>%	Relative Height<br>%	Amount<br>n.a.
1		18,557	382,032	423,907	84,05	90,60	n.a.
2		33,030	72,515	44,003	15,95	9,40	n.a.
**Total:**			**454,547**	**467,911**	**100,00**	**100,00**	

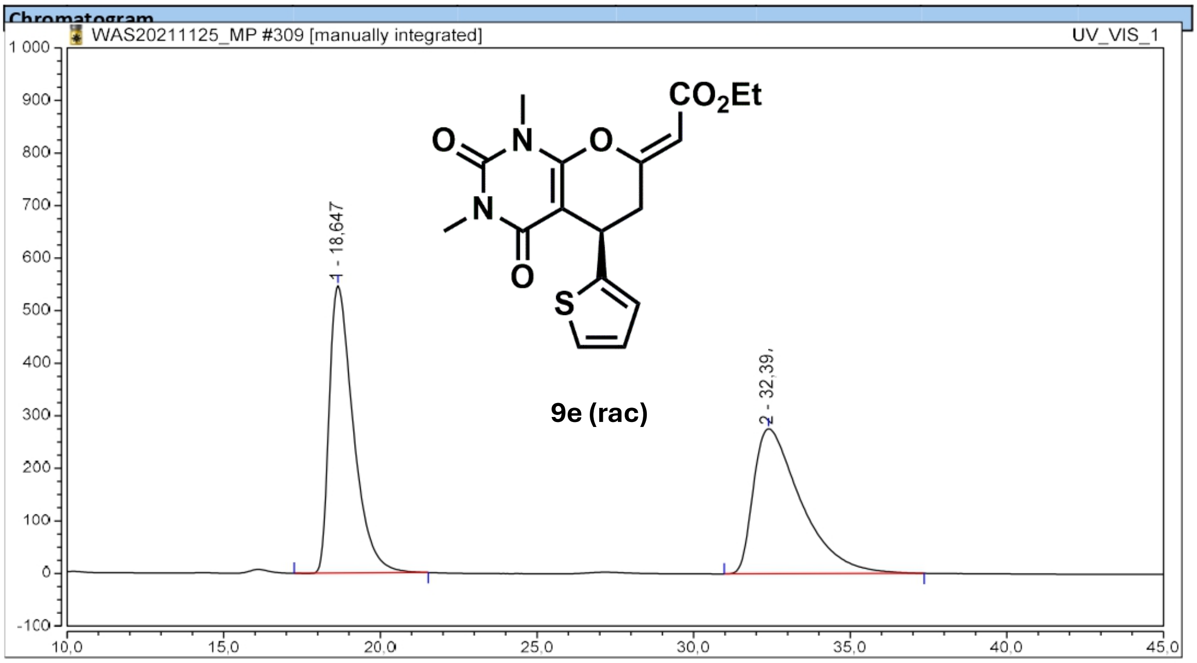

Integration Results							
No.	Peak Name	Retention Time min	Area mAU*min	Height mAU	Relative Area %	Relative Height %	Amount n.a.
1		18,647	487,626	546,722	50,04	66,46	n.a.
2		32,397	486,844	275,908	49,96	33,54	n.a.
**Total:**			**974,470**	**822,630**	**100,00**	**100,00**	

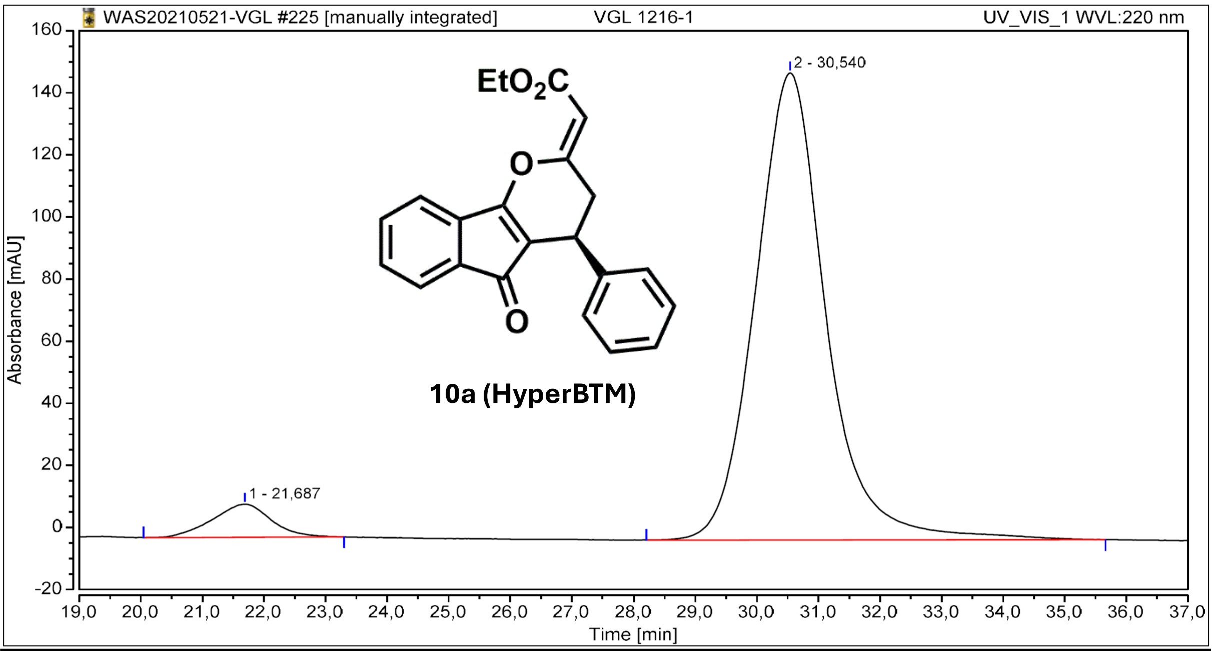

Integration Results							
No.	Peak Name	Retention Time min	Area mAU*min	Height mAU	Relative Area %	Relative Height %	Amount n.a.
1		21,687	11,803	10,652	5,58	6,61	n.a.
2		30,540	199,737	150,426	94,42	93,39	n.a.
**Total:**			**211,540**	**161,078**	**100,00**	**100,00**	

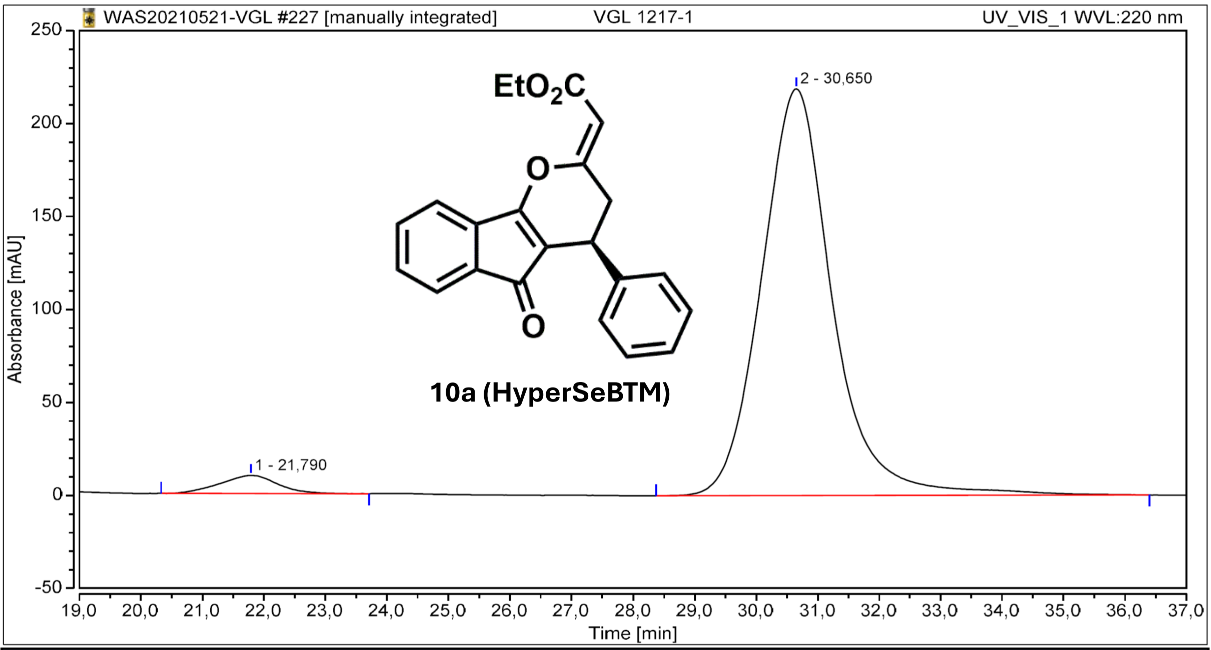

Integration Results							
No.	Peak Name	Retention Time min	Area mAU*min	Height mAU	Relative Area %	Relative Height %	Amount n.a.
1		21,790	10,492	9,726	3,47	4,26	n.a.
2		30,650	292,096	218,750	96,53	95,74	n.a.
**Total:**			**302,589**	**228,476**	**100,00**	**100,00**	

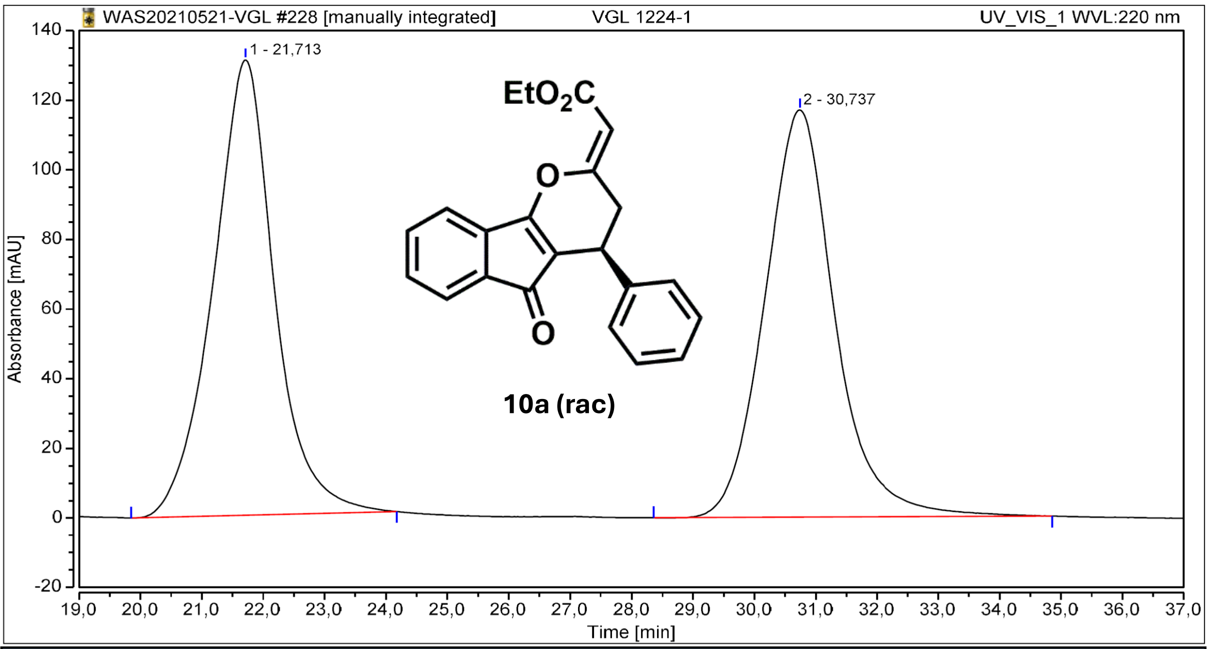

Integration Results							
No.	Peak Name	Retention Time min	Area mAU*min	Height mAU	Relative Area %	Relative Height %	Amount n.a.
1		21,713	151,603	130,791	49,54	52,79	n.a.
2		30,737	154,414	116,951	50,46	47,21	n.a.
**Total:**			**306,017**	**247,742**	**100,00**	**100,00**	

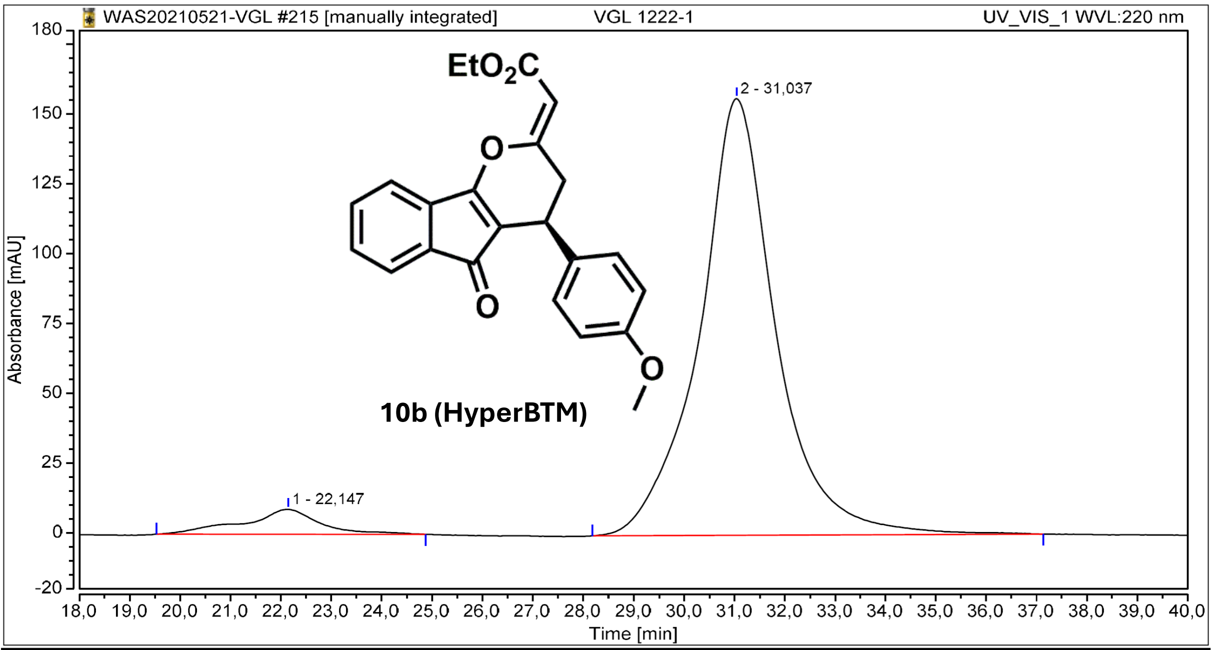

Integration Results							
No.	Peak Name	Retention Time min	Area mAU*min	Height mAU	Relative Area %	Relative Height %	Amount n.a.
1		22,147	15,742	8,894	5,44	5,38	n.a.
2		31,037	273,886	156,388	94,56	94,62	n.a.
**Total:**			**289,628**	**165,282**	**100,00**	**100,00**	

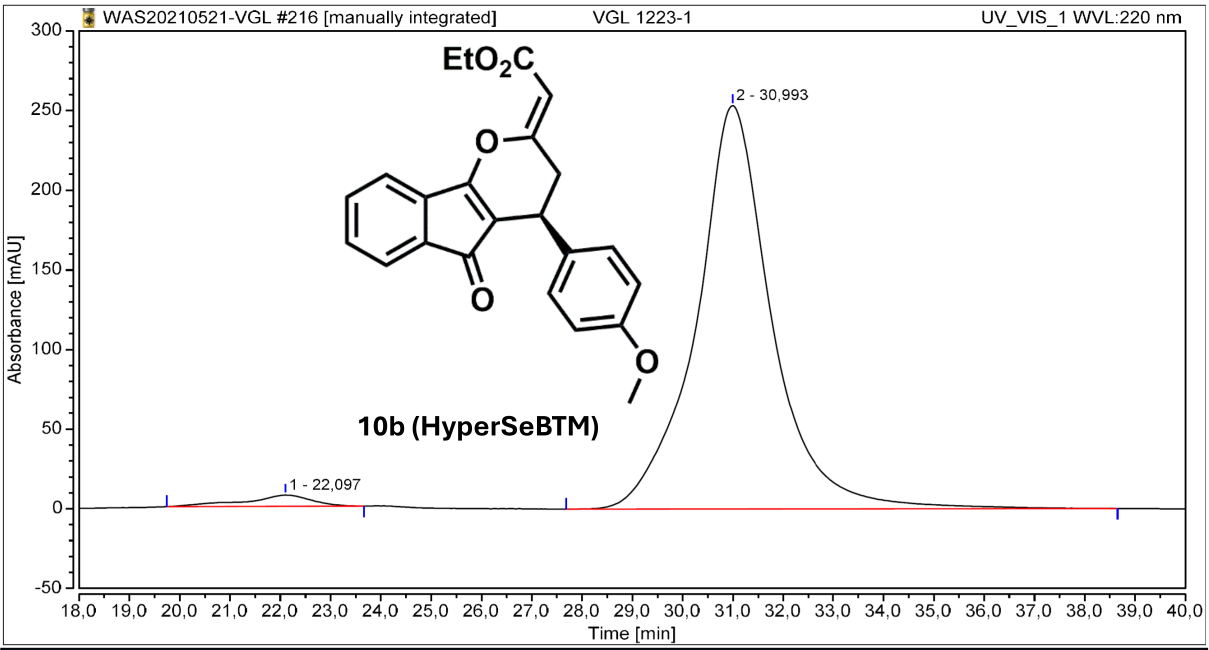

Integration Results							
No.	Peak Name	Retention Time min	Area mAU*min	Height mAU	Relative Area %	Relative Height %	Amount n.a.
1		22,097	10,711	7,032	2,37	2,70	n.a.
2		30,993	440,897	253,232	97,63	97,30	n.a.
**Total:**			**451,608**	**260,264**	**100,00**	**100,00**	

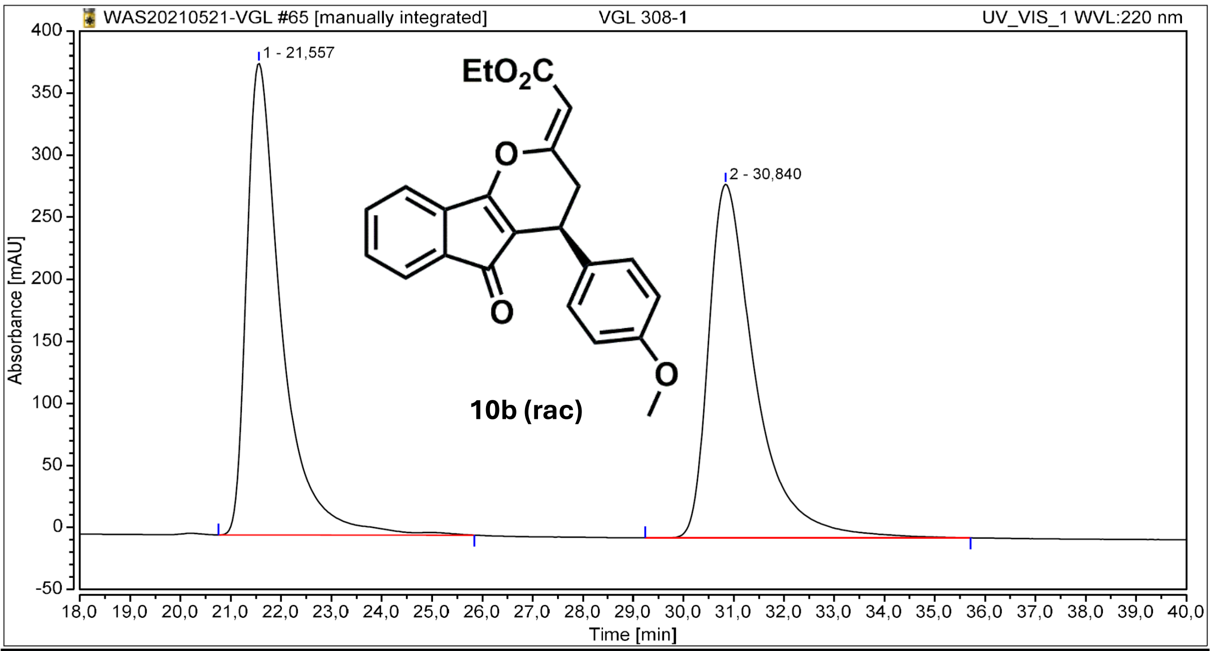

Integration Results							
No.	Peak Name	Retention Time min	Area mAU*min	Height mAU	Relative Area %	Relative Height %	Amount n.a.
1		21,557	306,560	380,376	50,15	57,17	n.a.
2		30,840	304,720	284,944	49,85	42,83	n.a.
**Total:**			**611,280**	**665,320**	**100,00**	**100,00**	

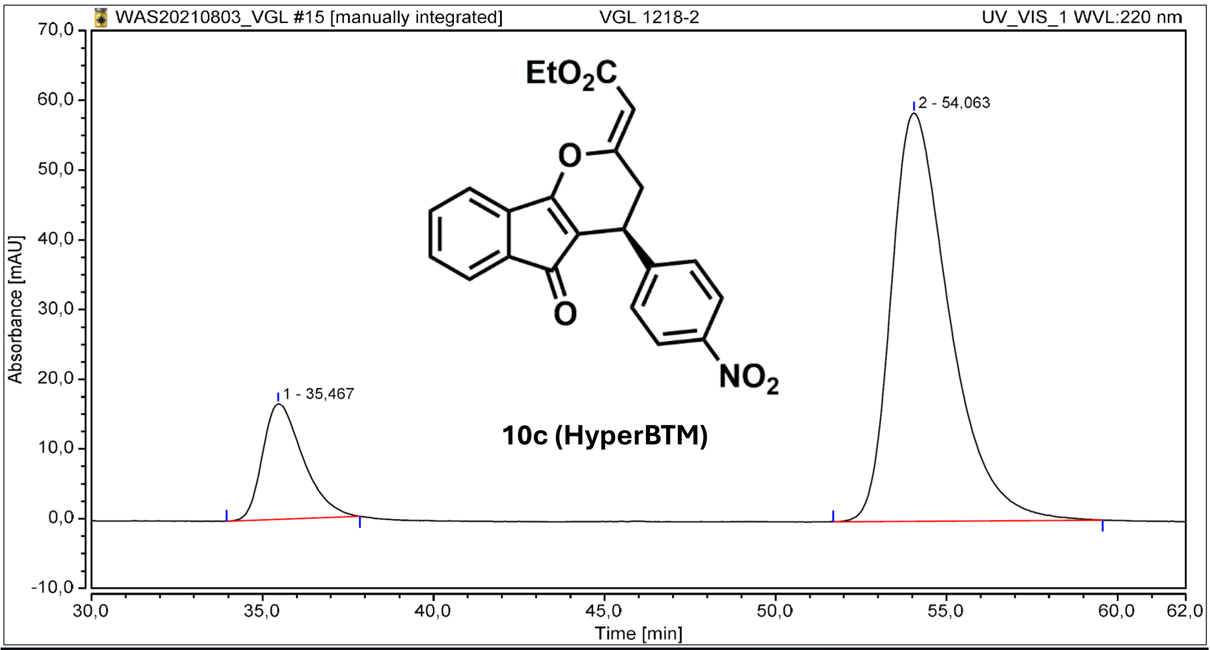

Integration Results							
No.	Peak Name	Retention Time min	Area mAU*min	Height mAU	Relative Area %	Relative Height %	Amount n.a.
1		35,467	22,731	16,585	16,43	22,07	n.a.
2		54,063	115,630	58,563	83,57	77,93	n.a.
**Total:**			**138,361**	**75,147**	**100,00**	**100,00**	

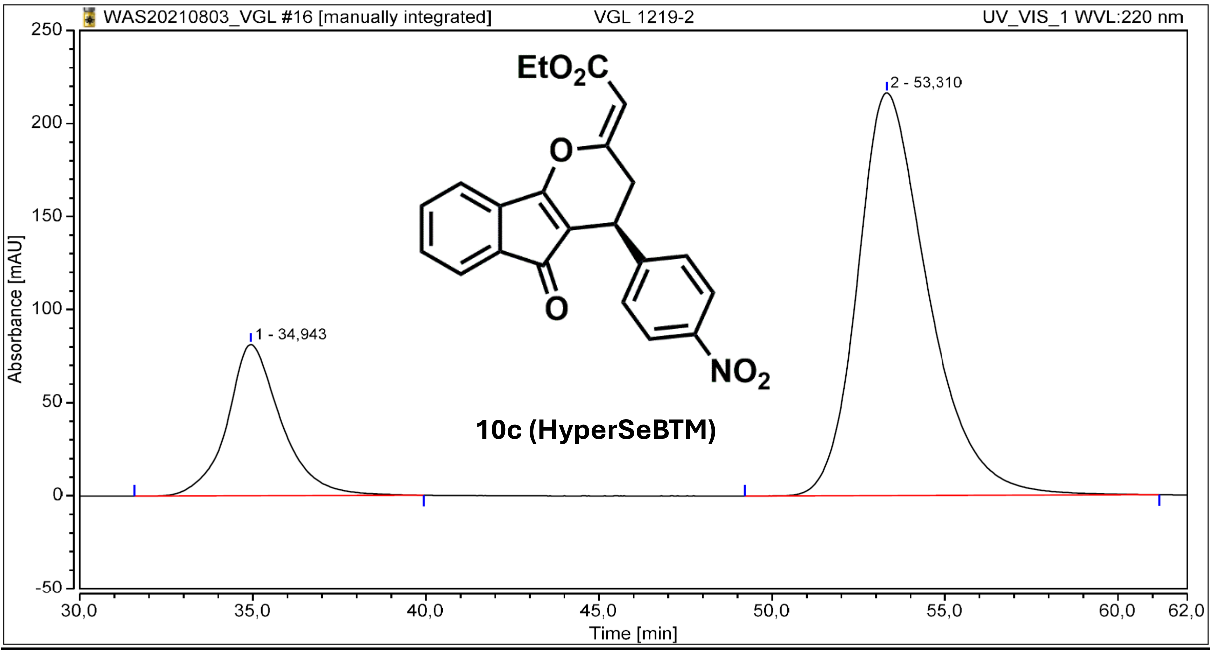

Integration Results							
No.	Peak Name	Retention Time min	Area mAU*min	Height mAU	Relative Area %	Relative Height %	Amount n.a.
1		34,943	146,937	81,232	22,24	27,30	n.a.
2		53,310	513,832	216,347	77,76	72,70	n.a.
**Total:**			**660,769**	**297,579**	**100,00**	**100,00**	

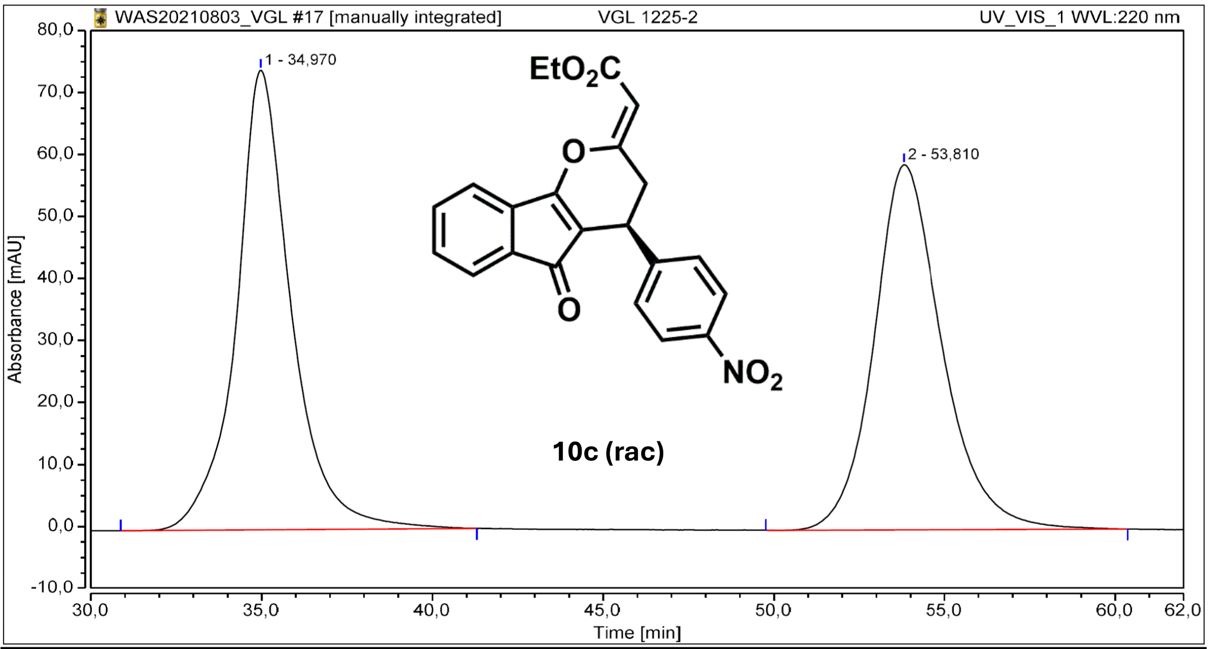

Integration Results							
No.	Peak Name	Retention Time min	Area mAU*min	Height mAU	Relative Area %	Relative Height %	Amount n.a.
1		34,970	138,572	74,138	50,31	55,74	n.a.
2		53,810	136,844	58,875	49,69	44,26	n.a.
**Total:**			**275,415**	**133,013**	**100,00**	**100,00**	

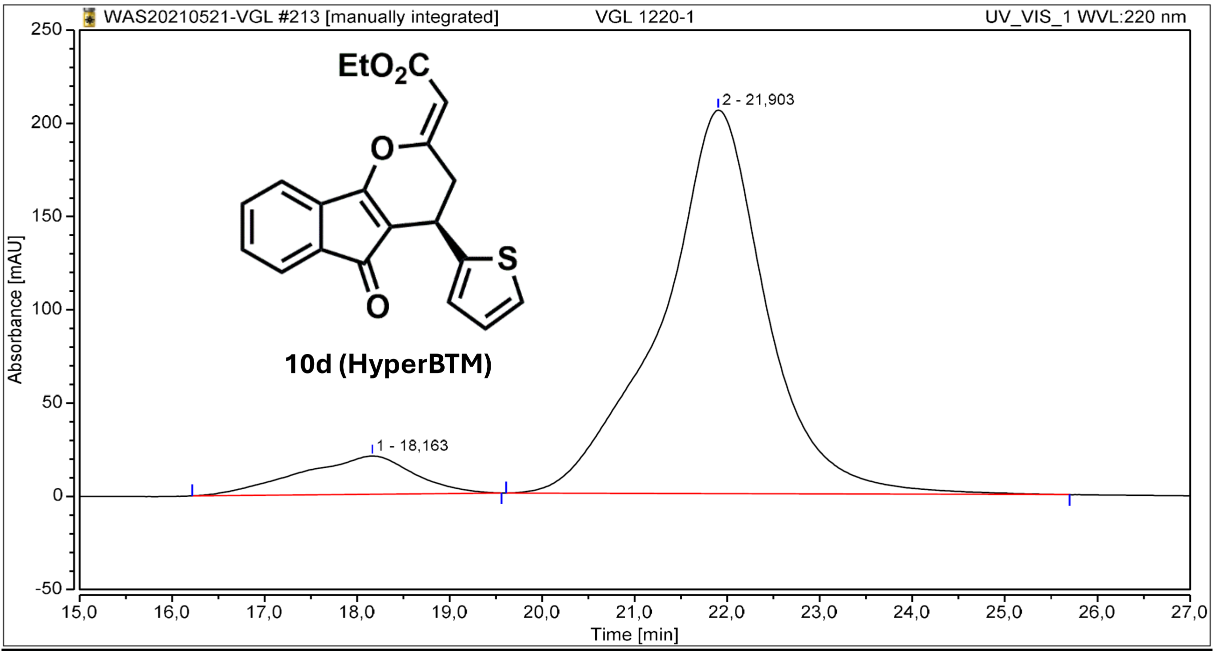

Integration Results							
No.	Peak Name	Retention Time min	Area mAU*min	Height mAU	Relative Area %	Relative Height %	Amount n.a.
1		18,163	28,893	20,438	9,69	9,04	n.a.
2		21,903	269,426	205,714	90,31	90,96	n.a.
**Total:**			**298,320**	**226,152**	**100,00**	**100,00**	

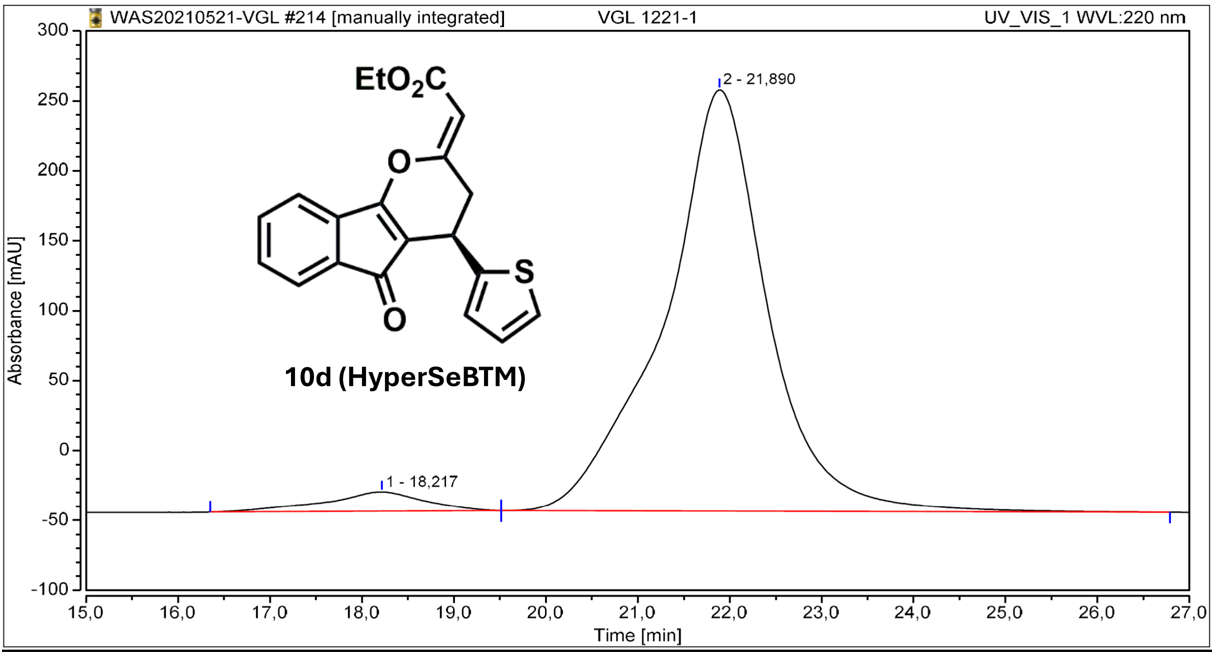

Integration Results							
No.	Peak Name	Retention Time min	Area mAU*min	Height mAU	Relative Area %	Relative Height %	Amount n.a.
1		18,217	17,296	13,848	4,22	4,39	n.a.
2		21,890	392,889	301,300	95,78	95,61	n.a.
**Total:**			**410,185**	**315,148**	**100,00**	**100,00**	

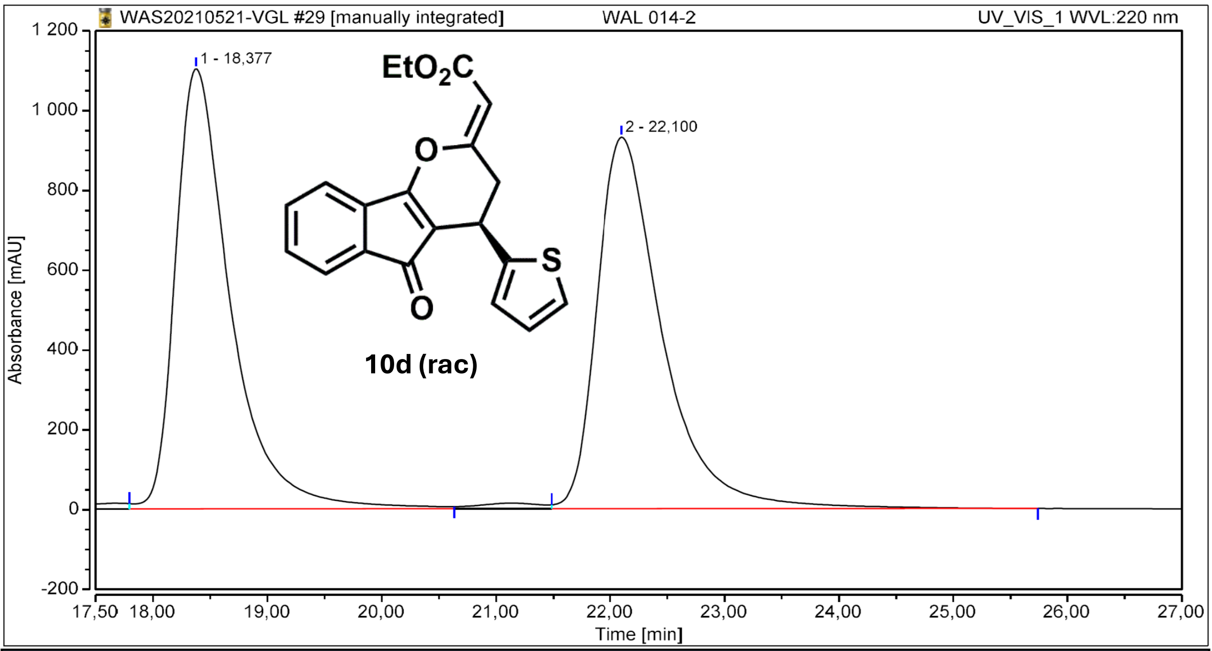

Integration Results							
No.	Peak Name	Retention Time min	Area mAU*min	Height mAU	Relative Area %	Relative Height %	Amount n.a.
1		18,377	616,082	1103,389	49,96	54,23	n.a.
2		22,100	617,161	931,360	50,04	45,77	n.a.
**Total:**			**1233,243**	**2034,749**	**100,00**	**100,00**	

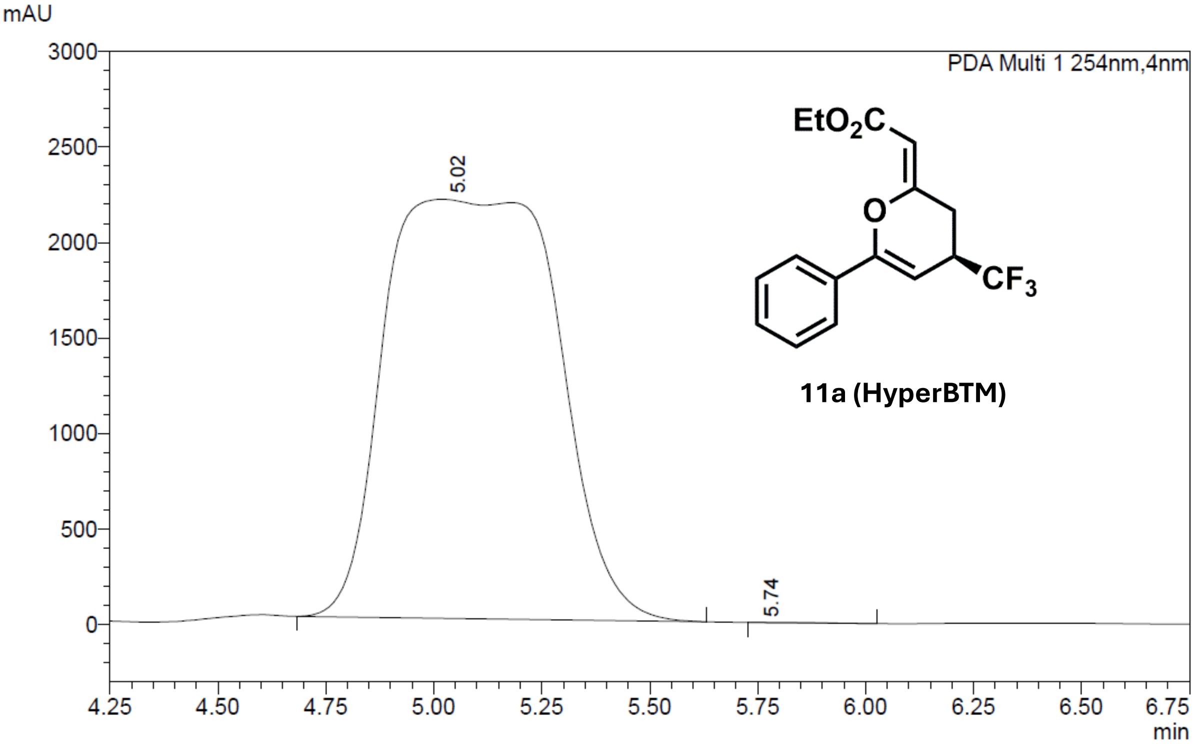

Peak Table

PDA Ch1 254nm

Peak#	Ret. Time	Area	Area%
1	5.02	60974576	99.99
2	5.74	6692	0.01
Total		60981268	100.00

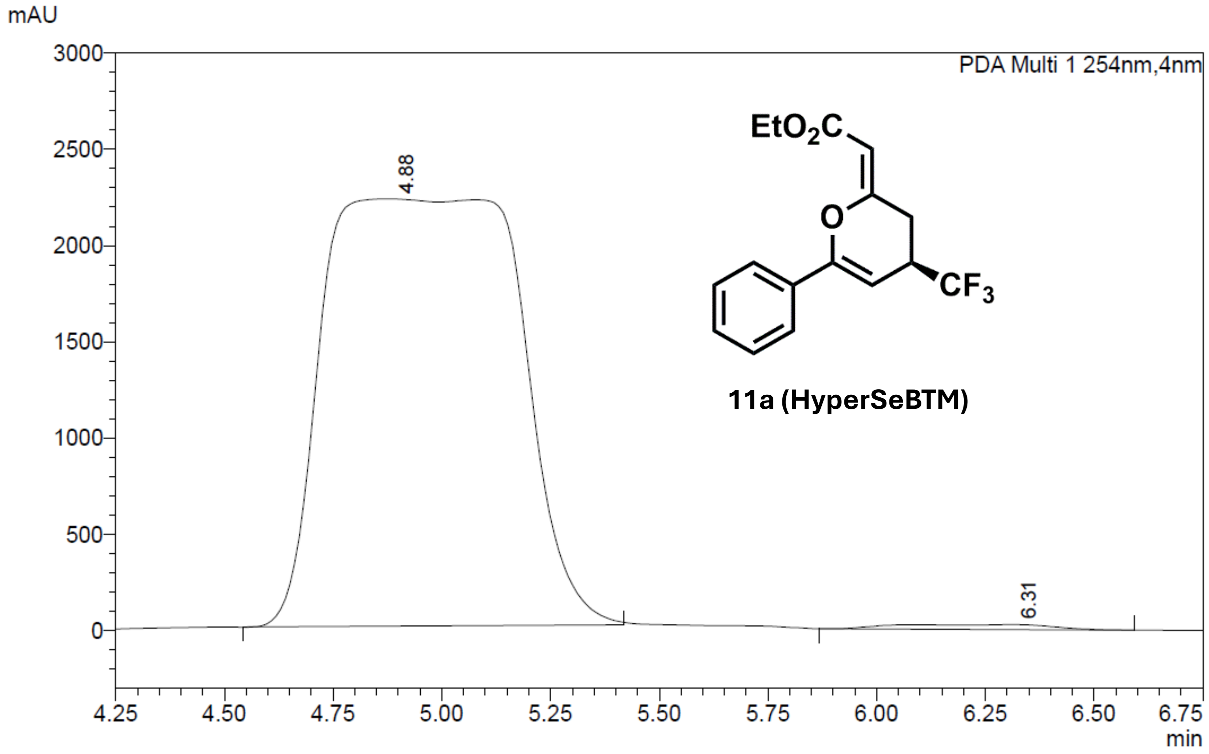

Peak Table

PDA Ch1 254nm

Peak#	Ret. Time	Area	Area%
1	4.88	69057087	99.08
2	6.31	640010	0.92
Total		69697096	100.00

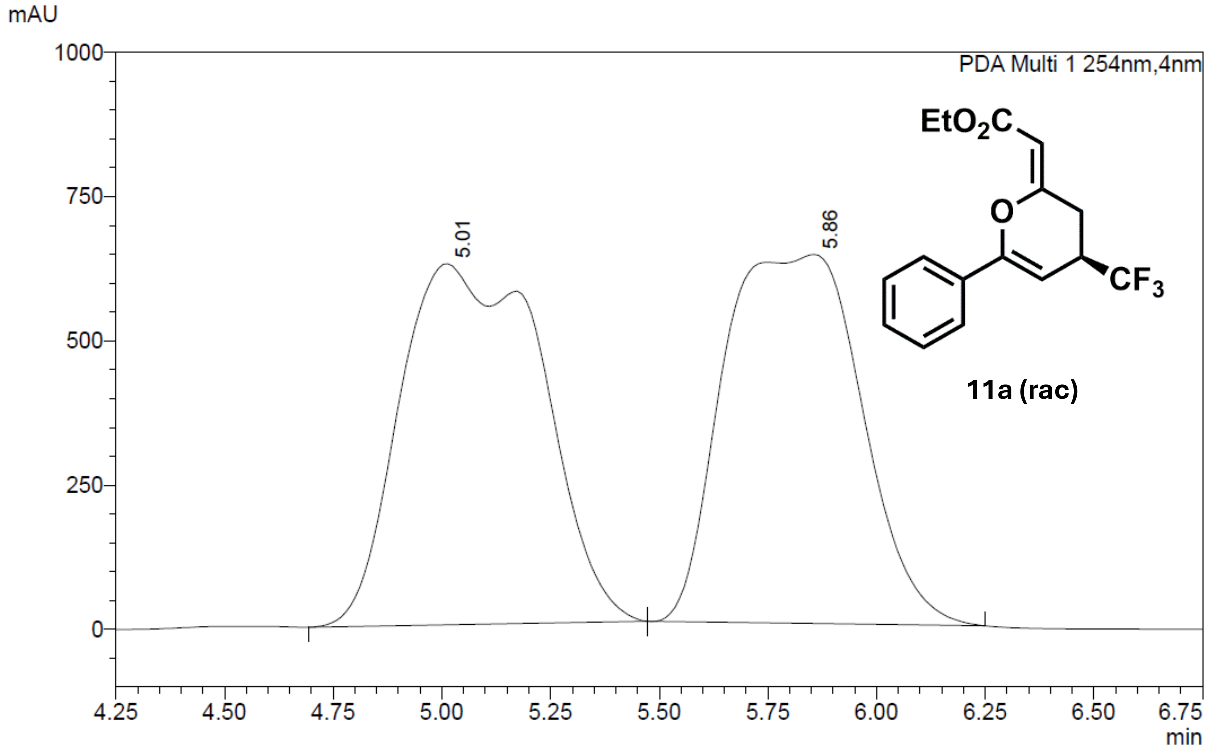

Peak Table

PDA Ch1 254nm

Peak#	Ret. Time	Area	Area%
1	5.01	14285150	50.26
2	5.86	14139658	49.74
Total		28424808	100.00

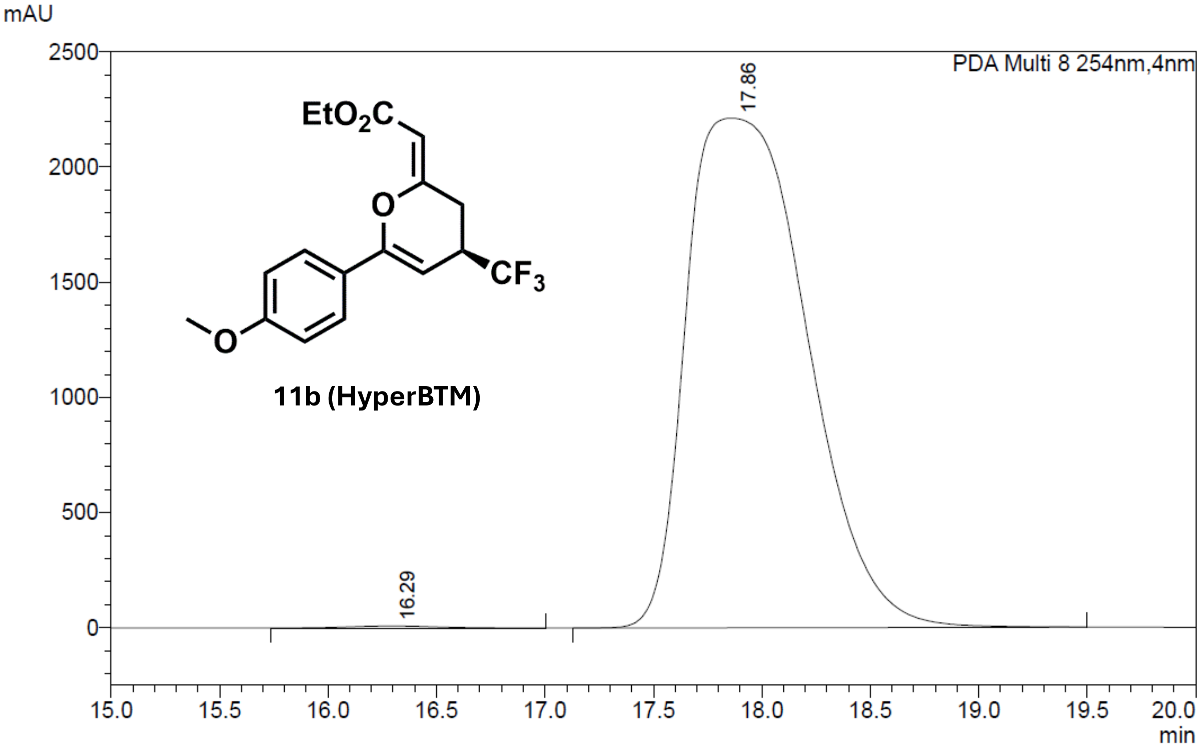

11b (HyperBTM)

Peak Table

PDA Ch8 254nm

Peak#	Ret. Time	Area	Area%
1	16.29	234979	0.27
2	17.86	85426099	99.73
Total		85661078	100.00

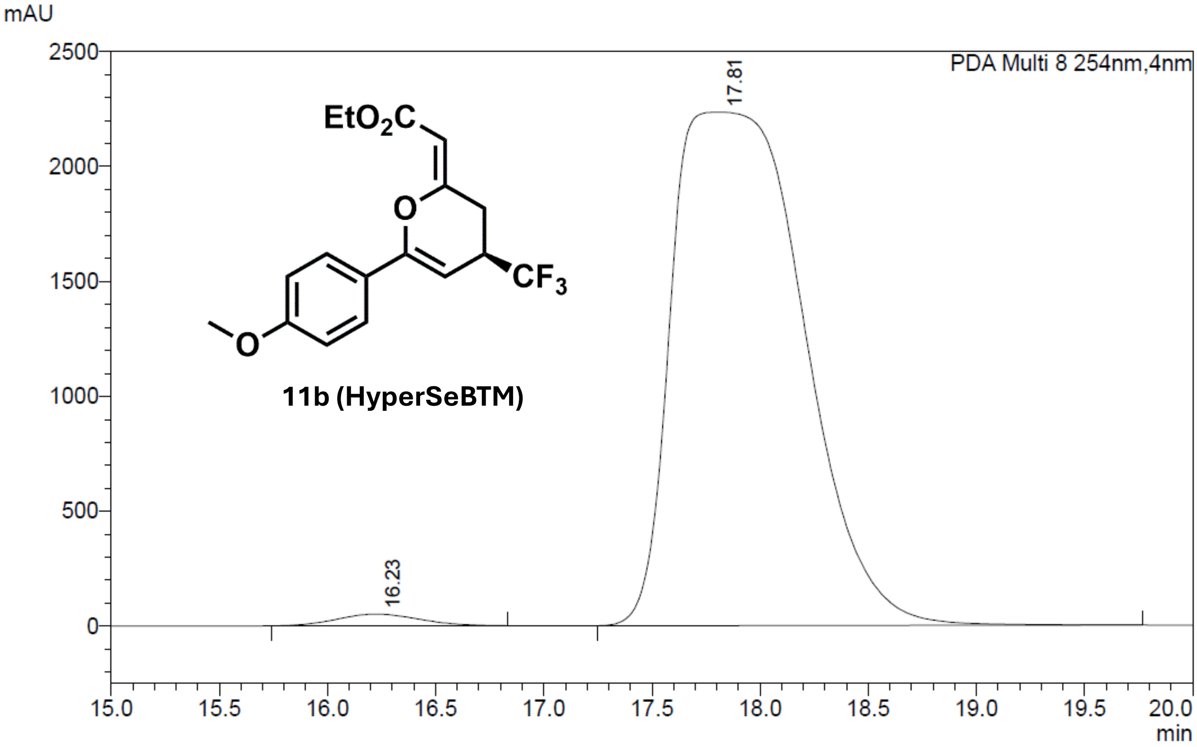

11b (HyperSeBTM)

Peak Table

PDA Ch8 254nm

Peak#	Ret. Time	Area	Area%
1	16.23	1350394	1.41
2	17.81	94307675	98.59
Total		95658069	100.00

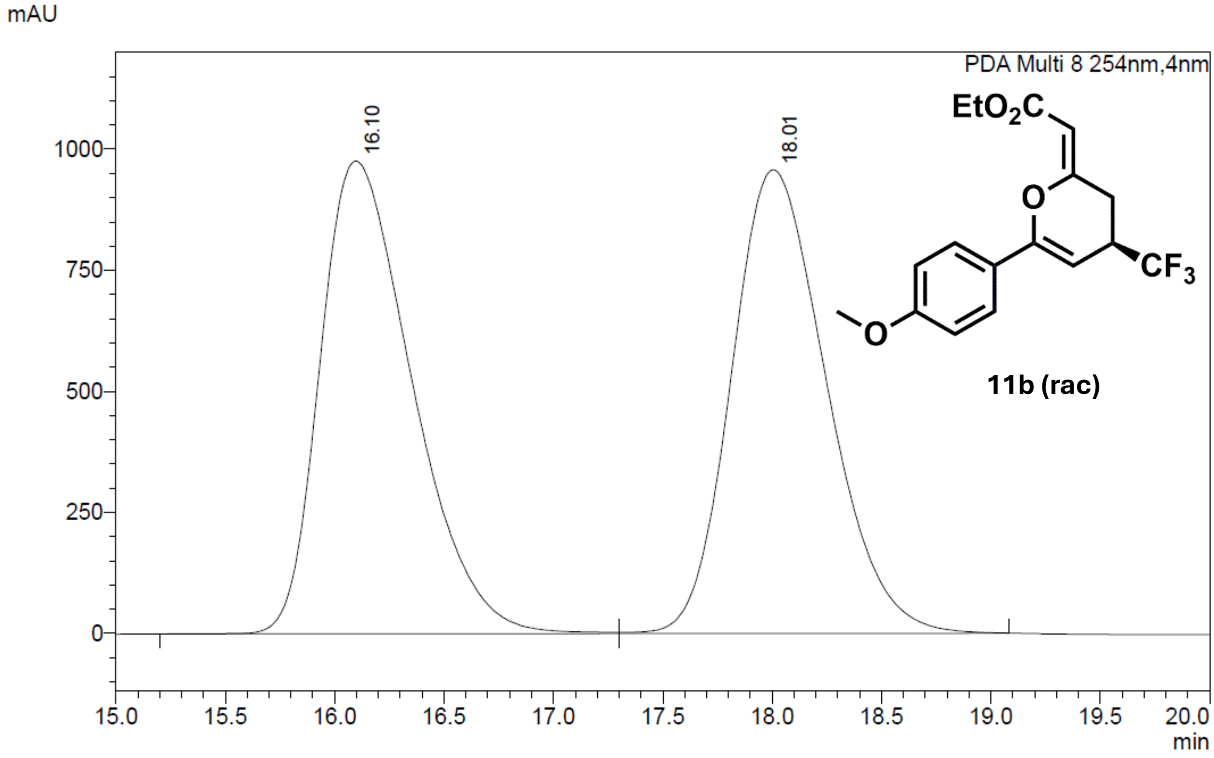

Peak Table

PDA Ch8 254nm

Peak#	Ret. Time	Area	Area%
1	16.10	28552597	49.68
2	18.01	28915187	50.32
Total		57467785	100.00

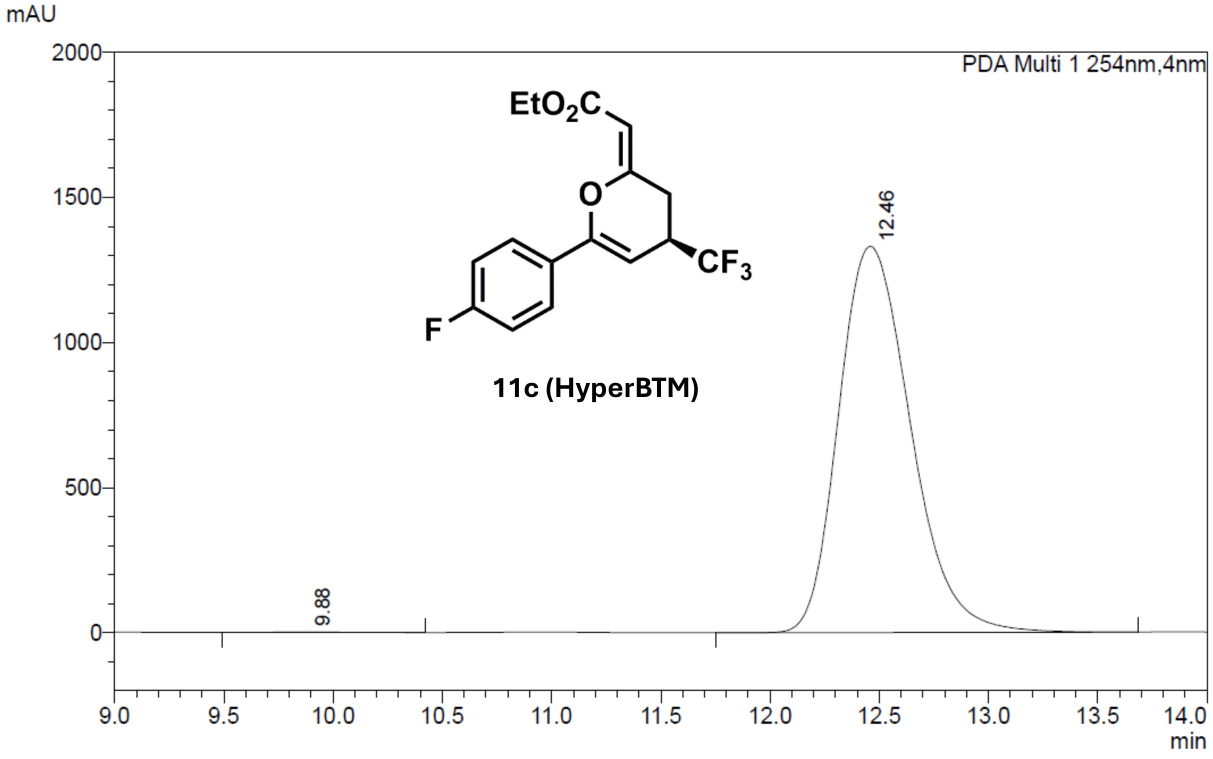

Peak Table

PDA Ch1 254nm

Peak#	Ret. Time	Area	Area%
1	9.88	70520	0.23
2	12.46	30472007	99.77
Total		30542527	100.00

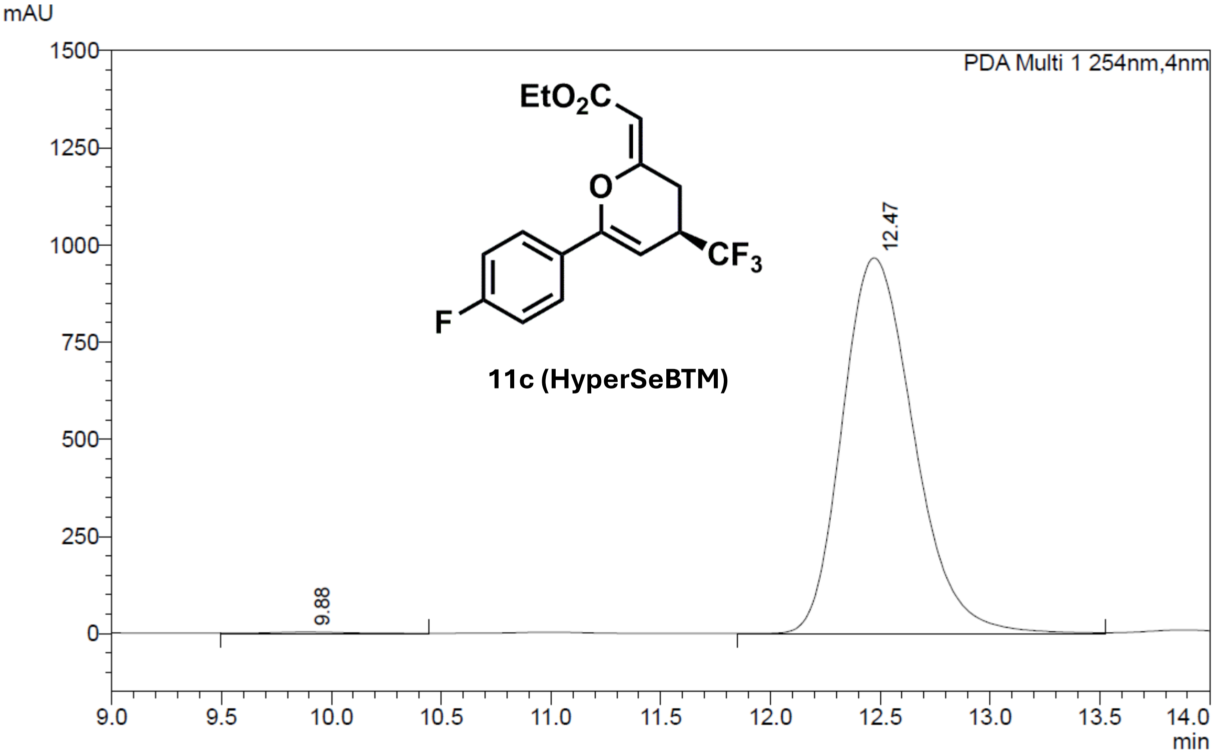

Peak Table

PDA Ch1 254nm

Peak#	Ret. Time	Area	Area%
1	9.88	85838	0.39
2	12.47	21903476	99.61
Total		21989314	100.00

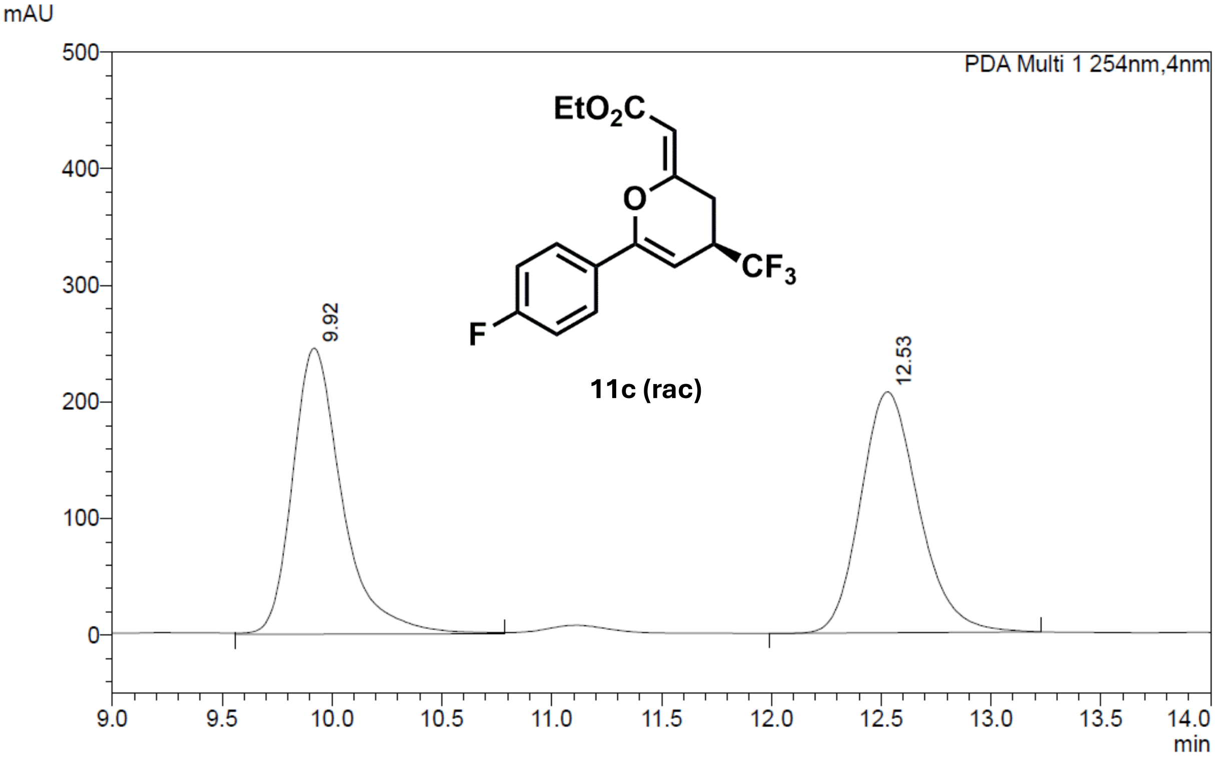

Peak Table

PDA Ch1 254nm

Peak#	Ret. Time	Area	Area%
1	9.92	3857529	50.36
2	12.53	3801964	49.64
Total		7659493	100.00

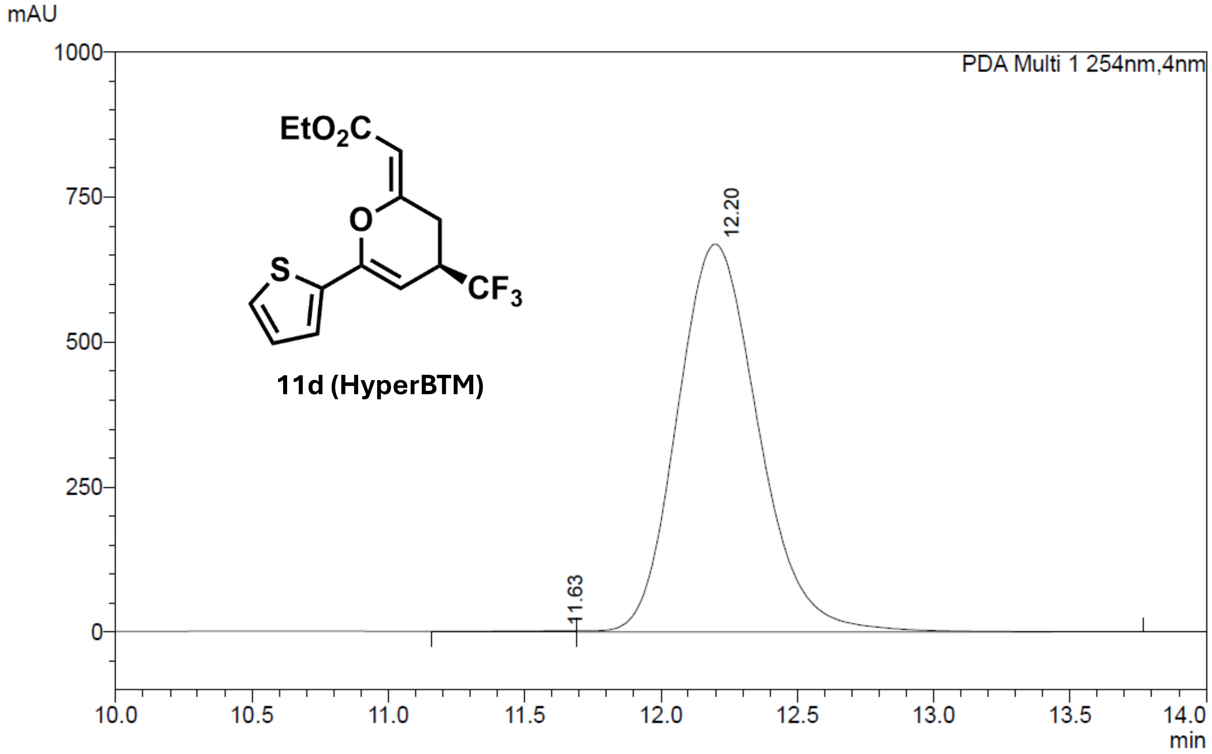

Peak Table

PDA Ch1 254nm

Peak#	Ret. Time	Area	Area%
1	11.63	41728	0.30
2	12.20	13853896	99.70
Total		13895624	100.00

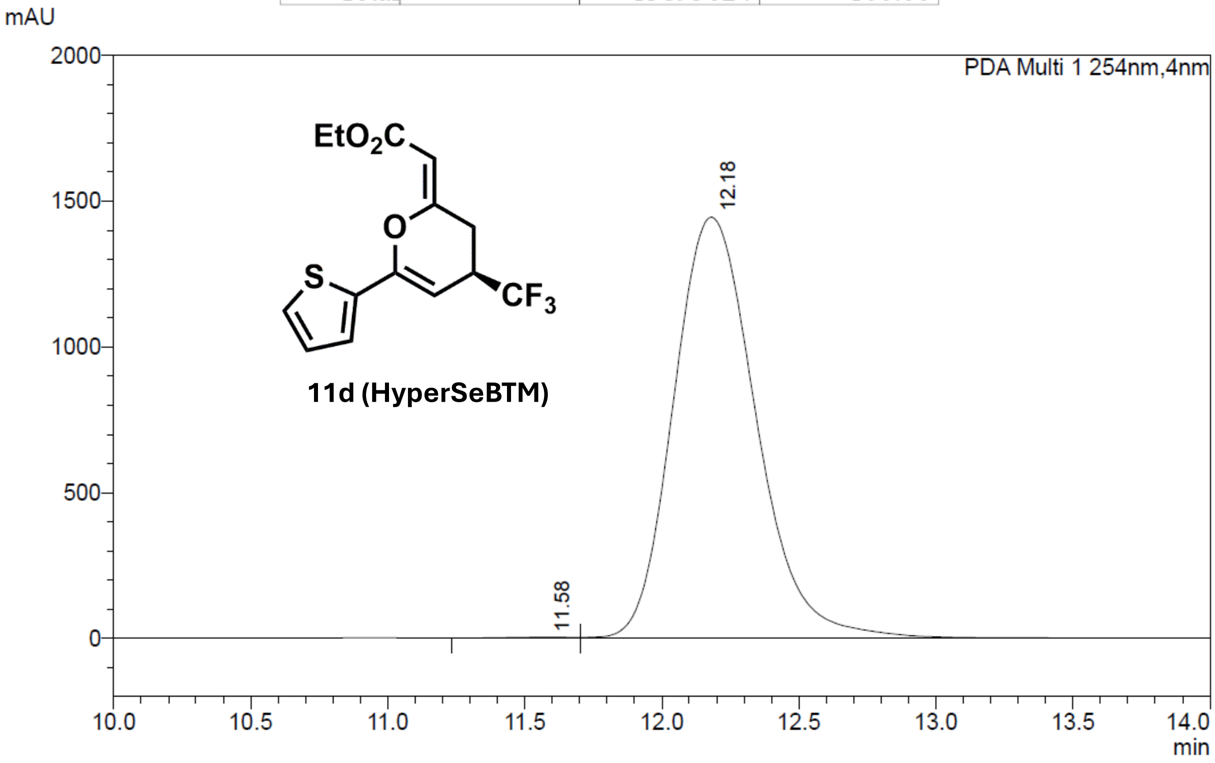

Peak Table

PDA Ch1 254nm

Peak#	Ret. Time	Area	Area%
1	11.58	56014	0.18
2	12.18	30260117	99.82
Total		30316131	100.00

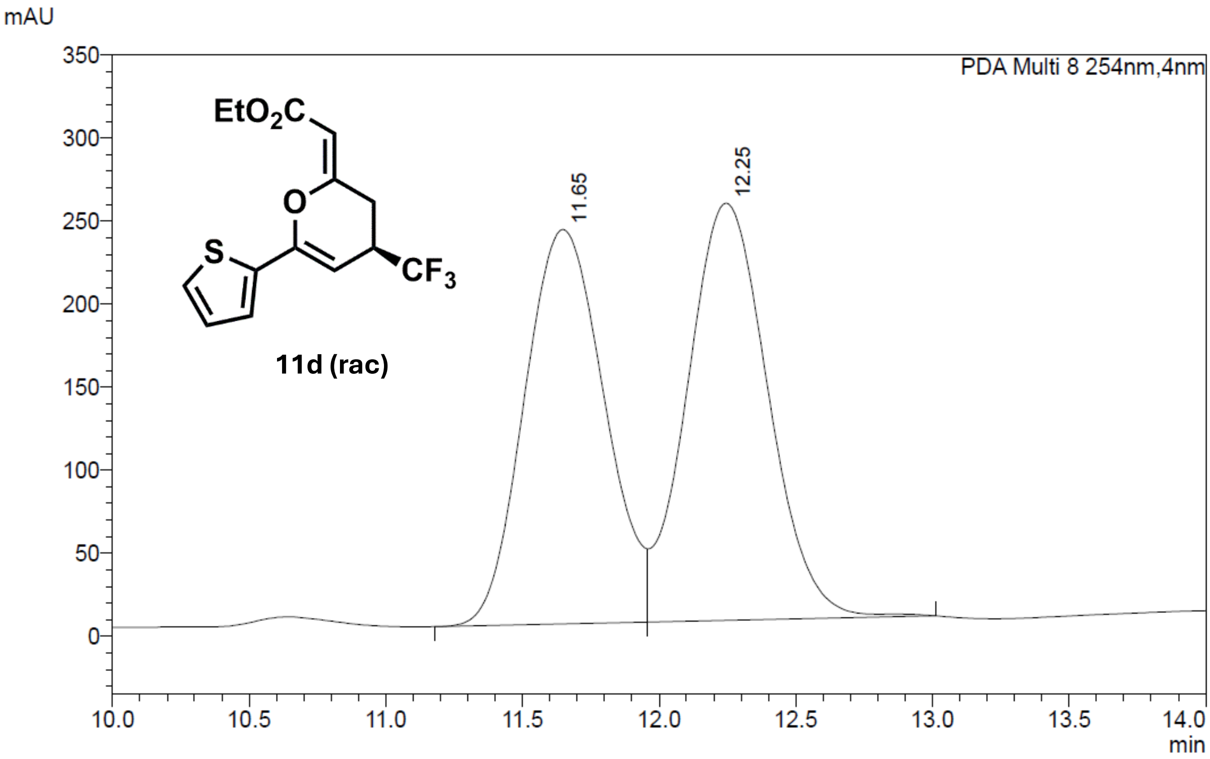

Peak Table

PDA Ch8 254nm

Peak#	Ret. Time	Area	Area%
1	11.65	4852926	48.35
2	12.25	5183171	51.65
Total		10036096	100.00

Chromatogram

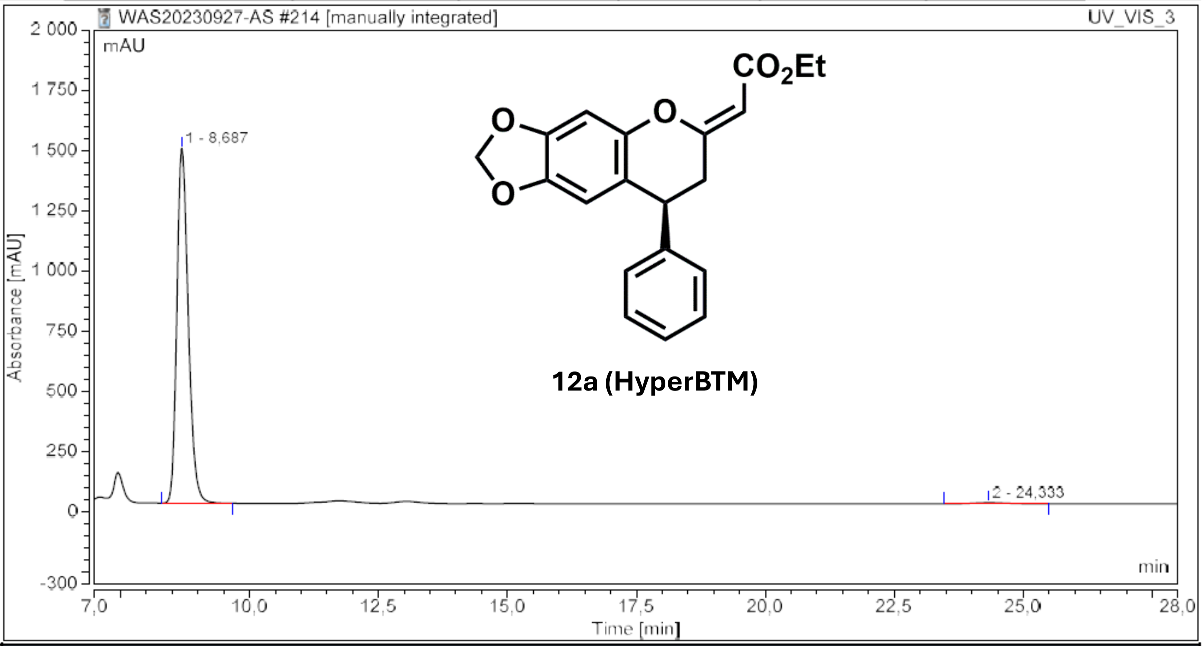

Integration Results

No.	Peak Name	Retention Time min	Area mAU*min	Height mAU	Relative Area %	Relative Height %	Amount n.a.
1		8,687	379,250	1474,180	99,09	99,69	n.a.
2		24,333	3,497	4,550	0,91	0,31	n.a.
**Total:**			**382,747**	**1478,730**	**100,00**	**100,00**	

Chromatogram

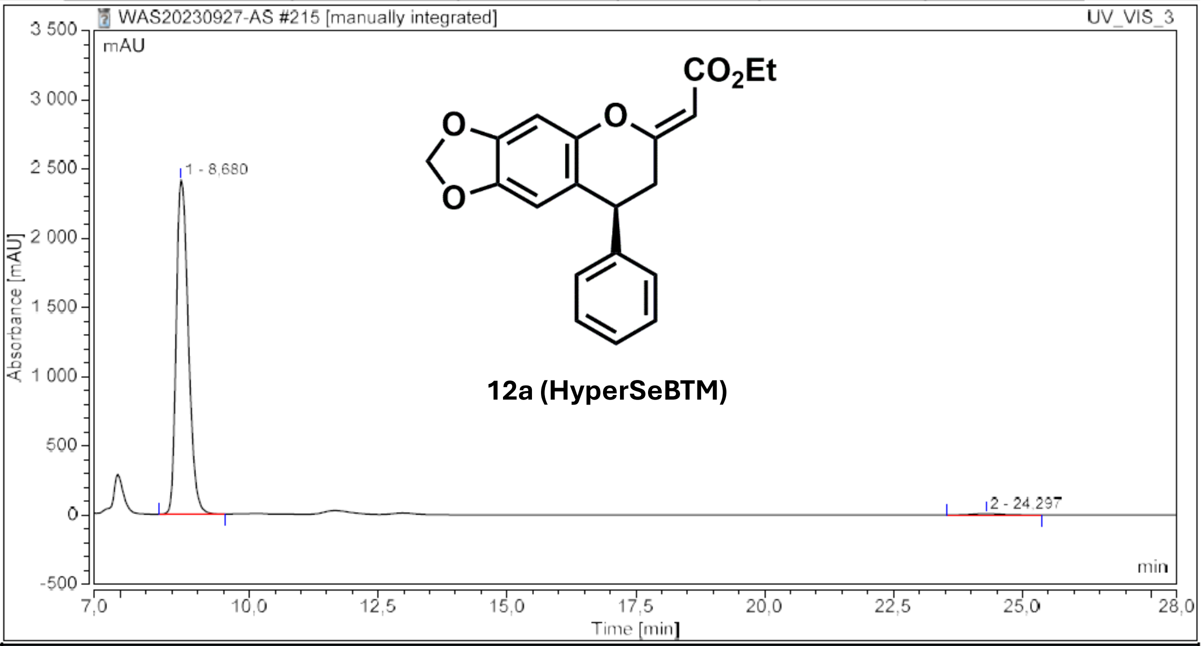

Integration Results

No.	Peak Name	Retention Time min	Area mAU*min	Height mAU	Relative Area %	Relative Height %	Amount n.a.
1		8,680	658,624	2414,767	98,82	99,57	n.a.
2		24,297	7,832	10,541	1,18	0,43	n.a.
**Total:**			**666,456**	**2425,307**	**100,00**	**100,00**	

Chromatogram

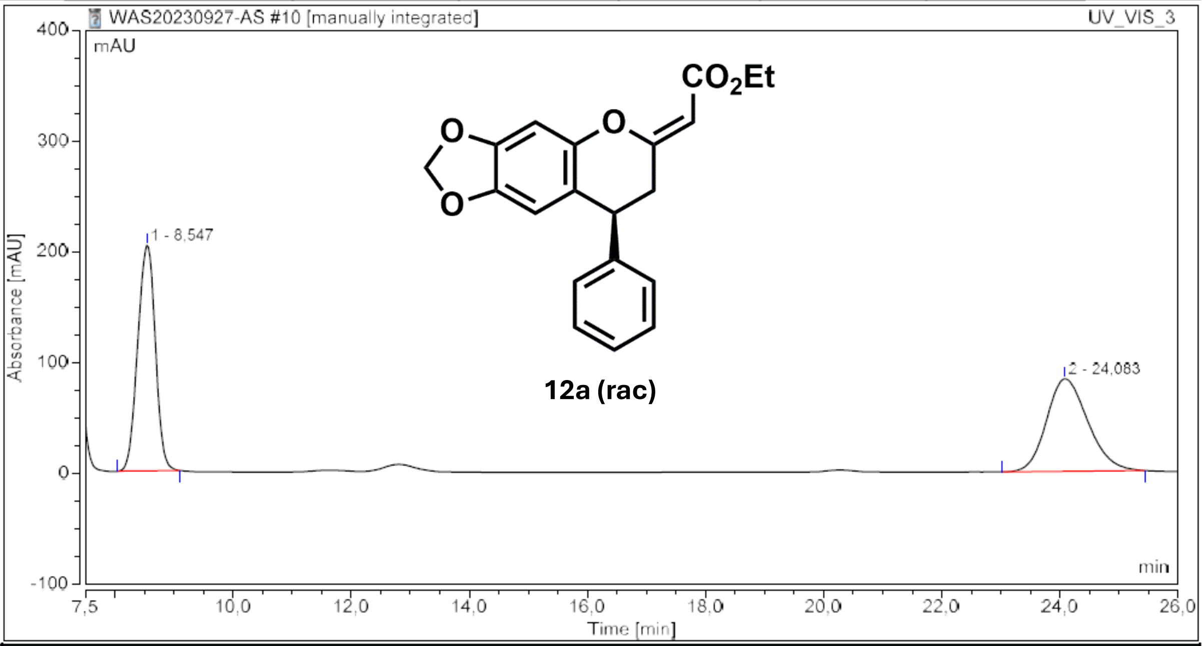

Integration Results

No.	Peak Name	Retention Time min	Area mAU*min	Height mAU	Relative Area %	Relative Height %	Amount n.a.
1		8,547	70,430	203,842	51,06	70,94	n.a.
2		24,083	67,515	83,489	48,94	29,06	n.a.
**Total:**			**137,944**	**287,331**	**100,00**	**100,00**	

Chromatogram

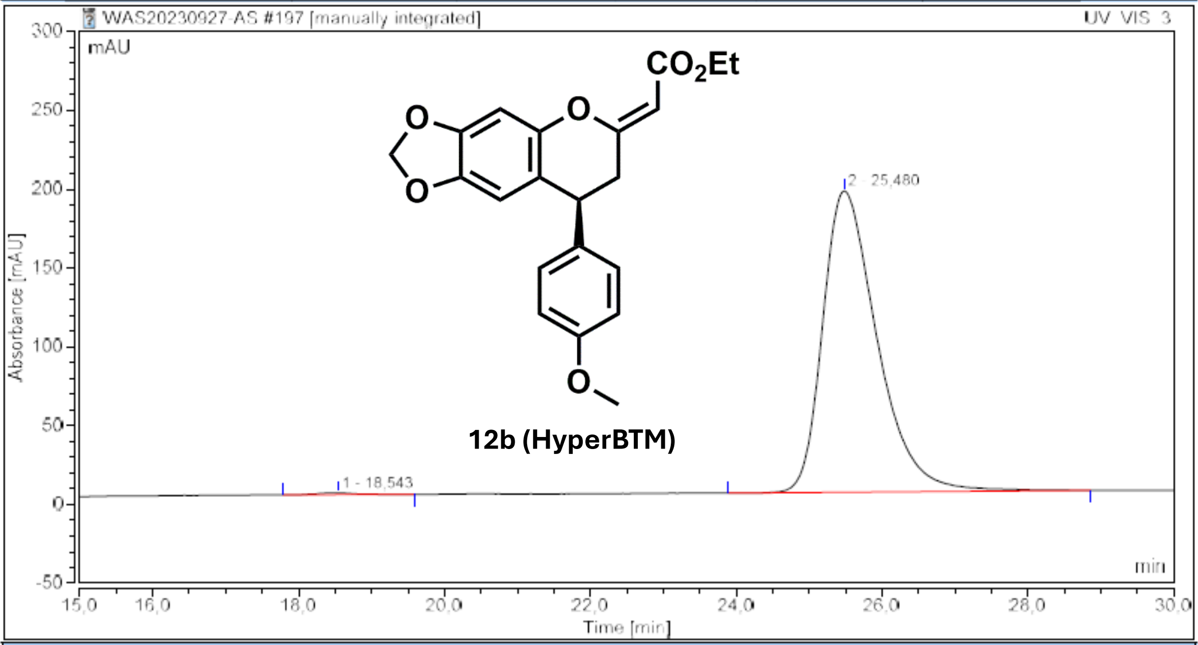

Integration Results

No.	Peak Name	Retention Time min	Area mAU*min	Height mAU	Relative Area %	Relative Height %	Amount n.a.
1		18,543	0,739	1,155	0,45	0,60	n.a.
2		25,480	165,110	191,092	99,55	99,40	n.a.
**Total:**			**165,849**	**192,247**	**100,00**	**100,00**	

Chromatogram

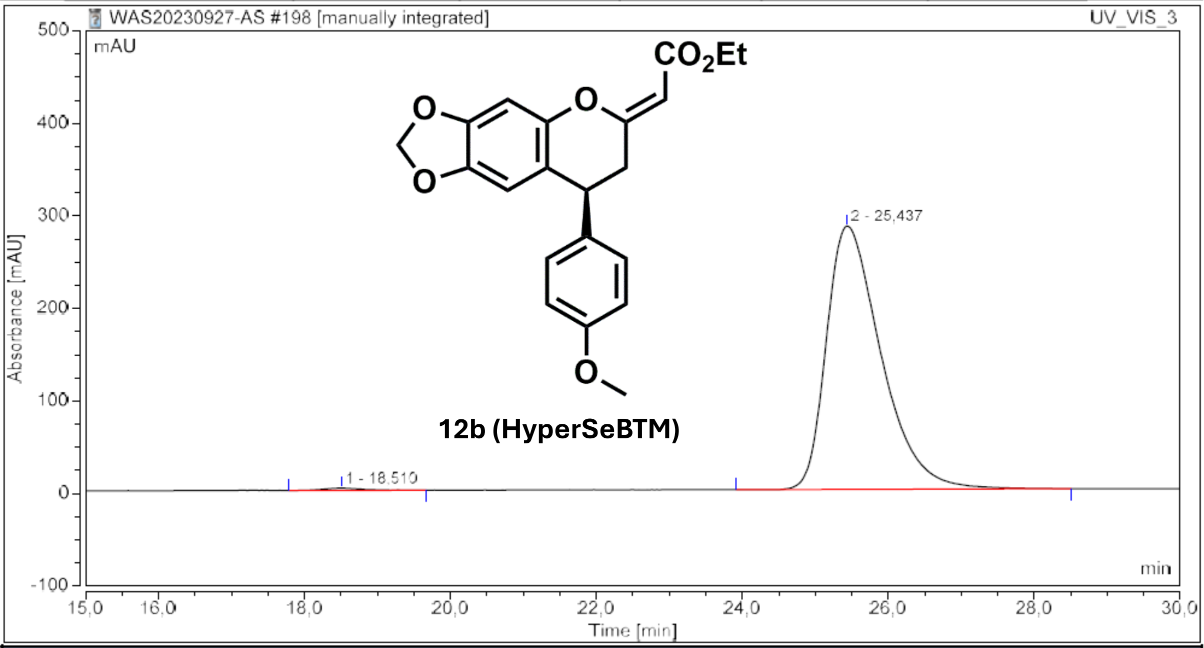

Integration Results

No.	Peak Name	Retention Time min	Area mAU*min	Height mAU	Relative Area %	Relative Height %	Amount n.a.
1		18,510	1,471	2,441	0,59	0,85	n.a.
2		25,437	246,616	284,644	99,41	99,15	n.a.
**Total:**			**248,087**	**287,085**	**100,00**	**100,00**	

Chromatogram

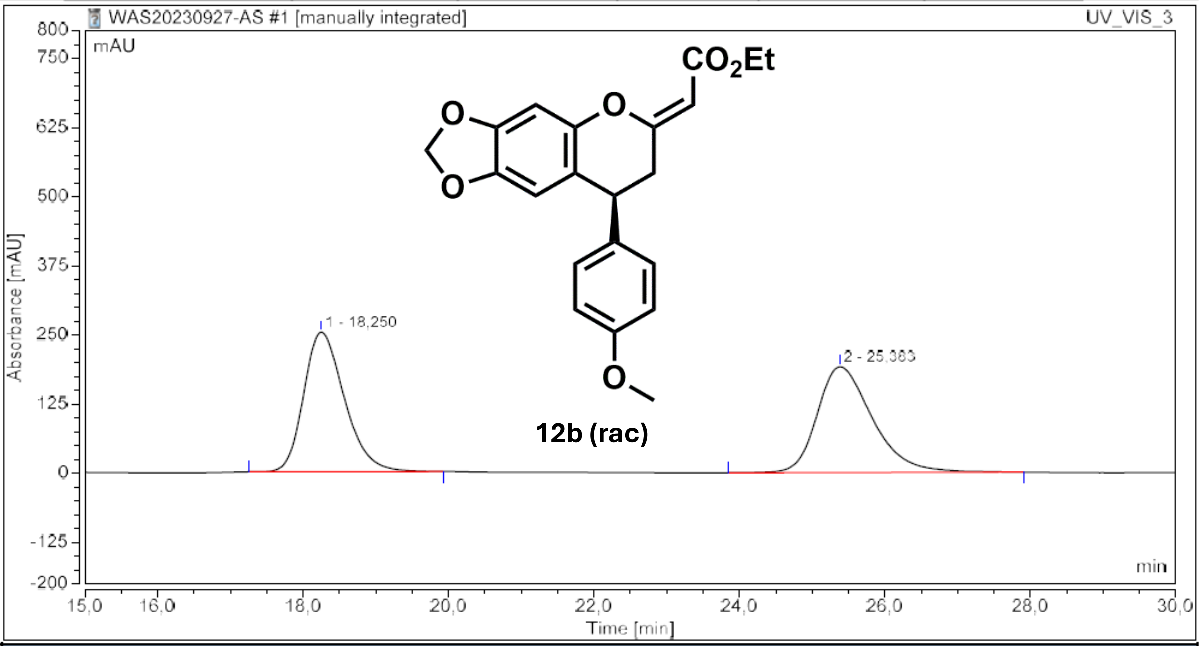

Integration Results

No.	Peak Name	Retention Time<br>min	Area<br>mAU*min	Height<br>mAU	Relative Area<br>%	Relative Height<br>%	Amount<br>n.a.
1		18,250	163,621	252,194	49,29	56,87	n.a.
2		25,383	168,315	191,262	50,71	43,13	n.a.
**Total:**			**331,936**	**443,456**	**100,00**	**100,00**	

Chromatogram

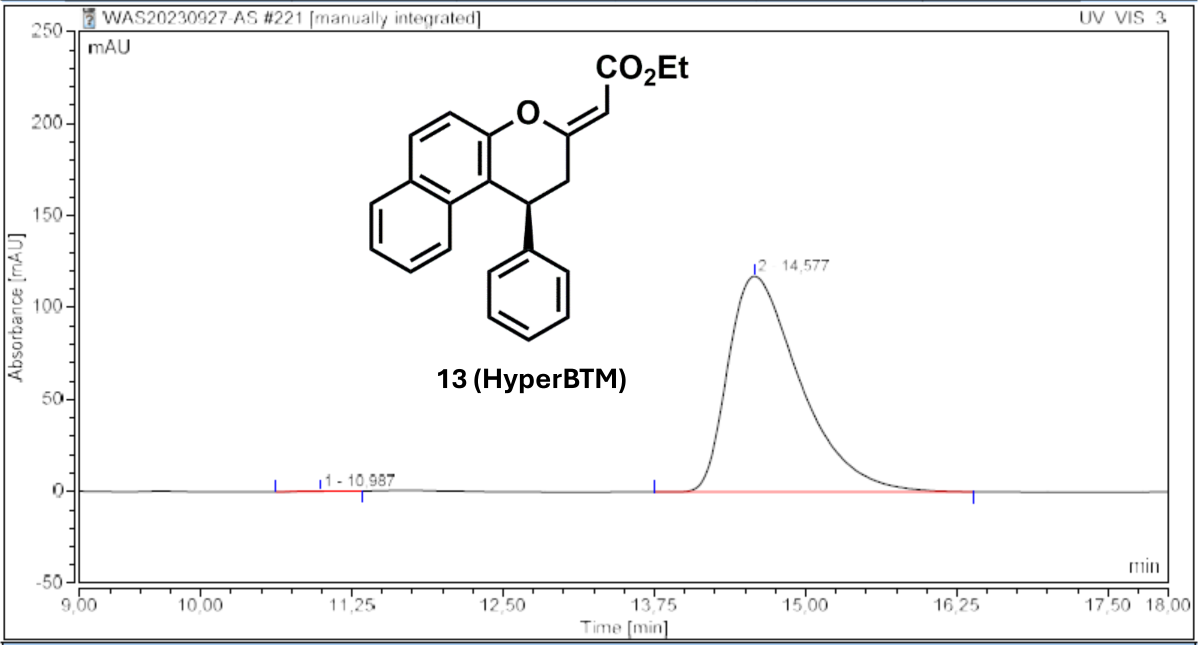

Integration Results

No.	Peak Name	Retention Time<br>min	Area<br>mAU*min	Height<br>mAU	Relative Area<br>%	Relative Height<br>%	Amount<br>n.a.
1		10,987	0,119	0,354	0,15	0,30	n.a.
2		14,577	81,192	117,277	99,85	99,70	n.a.
**Total:**			**81,311**	**117,630**	**100,00**	**100,00**	

Chromatogram

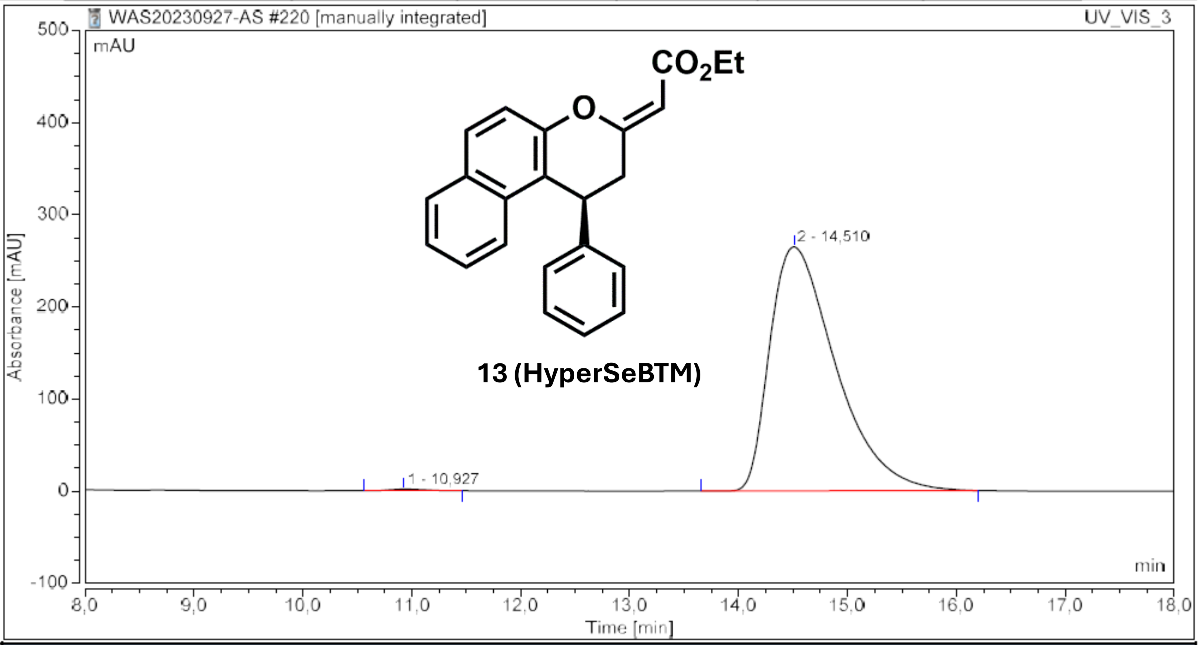

Integration Results

No.	Peak Name	Retention Time min	Area mAU*min	Height mAU	Relative Area %	Relative Height %	Amount n.a.
1		10,927	0,725	1,794	0,39	0,67	n.a.
2		14,510	185,556	264,834	99,61	99,33	n.a.
**Total:**			**186,281**	**266,629**	**100,00**	**100,00**	

Chromatogram

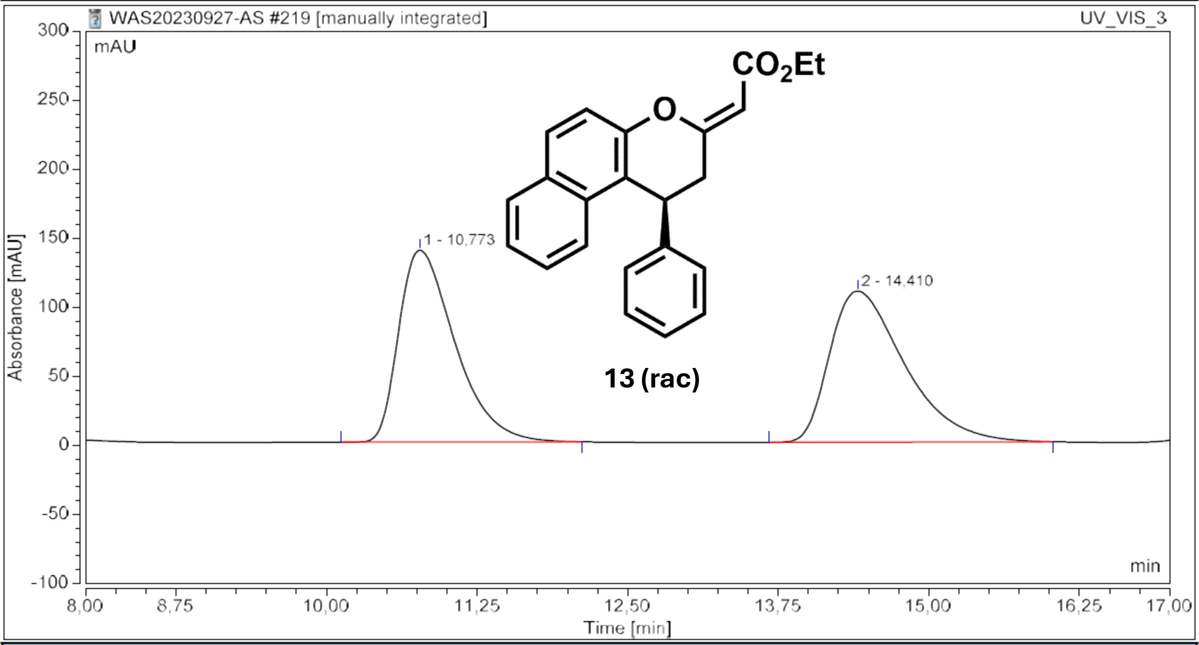

Integration Results

No.	Peak Name	Retention Time min	Area mAU*min	Height mAU	Relative Area %	Relative Height %	Amount n.a.
1		10,773	75,131	139,014	48,80	55,96	n.a.
2		14,410	78,813	109,388	51,20	44,04	n.a.
**Total:**			**153,945**	**248,402**	**100,00**	**100,00**	
